# Genomic Rearrangements and Functional Diversification of *lecA* and *lecB* Lectin-Coding Regions Impacting the Efficacy of Glycomimetics Directed against *Pseudomonas aeruginosa*

**DOI:** 10.3389/fmicb.2016.00811

**Published:** 2016-05-31

**Authors:** Amine M. Boukerb, Aude Decor, Sébastien Ribun, Rachel Tabaroni, Audric Rousset, Loris Commin, Samuel Buff, Anne Doléans-Jordheim, Sébastien Vidal, Annabelle Varrot, Anne Imberty, Benoit Cournoyer

**Affiliations:** ^1^Equipes de Recherche, Bactéries Pathogènes Opportunistes et Environnement, Centre de Ressources Biologiques - Environnement Microbiologie Lyon, UMR Centre National de la Recherche Scientifique 5557 Ecologie Microbienne, Université Lyon 1 and VetAgro SupLyon, France; ^2^Centre de Recherche sur les Macromolécules Végétales (UPR 5301), Centre National de la Recherche Scientifique and Université Grenoble AlpesGrenoble, France; ^3^Laboratoire de Chimie Organique 2 – Glycochimie, Institut de Chimie et Biochimie Moléculaires et Supramoléculaires, UMR Centre National de la Recherche Scientifique 5246, Université Lyon 1Lyon, France; ^4^Université de Lyon, VetAgro Sup, UPSP 2011-03-101, Interactions Cellules Environnement and CRB-ANIM (ANR-INBS11-0003)Marcy-L'Etoile, France

**Keywords:** *Pseudomonas aeruginosa*, soluble lectins, PA7 clade, region of genomic plasticity (RGP), glycoclusters, flow cytometry, glycan array, crystallography

## Abstract

LecA and LecB tetrameric lectins take part in oligosaccharide-mediated adhesion-processes of *Pseudomonas aeruginosa*. Glycomimetics have been designed to block these interactions. The great versatility of *P. aeruginosa* suggests that the range of application of these glycomimetics could be restricted to genotypes with particular lectin types. The likelihood of having genomic and genetic changes impacting LecA and LecB interactions with glycomimetics such as galactosylated and fucosylated calix[4]arene was investigated over a collection of strains from the main clades of *P. aeruginosa*. Lectin types were defined, and their ligand specificities were inferred. These analyses showed a loss of *lecA* among the PA7 clade. Genomic changes impacting *lec* loci were thus assessed using strains of this clade, and by making comparisons with the PAO1 genome. The *lecA* regions were found challenged by phage attacks and PAGI-2 (genomic island) integrations. A prophage was linked to the loss of *lecA*. The *lecB* regions were found less impacted by such rearrangements but greater *lecB* than *lecA* genetic divergences were recorded. Sixteen combinations of LecA and LecB types were observed. Amino acid variations were mapped on PAO1 crystal structures. Most significant changes were observed on LecB_PA7_, and found close to the fucose binding site. Glycan array analyses were performed with purified LecB_PA7_. LecB_PA7_ was found less specific for fucosylated oligosaccharides than LecB_PAO1_, with a preference for H type 2 rather than type 1, and Lewis^a^ rather than Lewis^x^. Comparison of the crystal structures of LecB_PA7_ and LecB_PAO1_ in complex with Lewis^a^ showed these changes in specificity to have resulted from a modification of the water network between the lectin, galactose and GlcNAc residues. Incidence of these modifications on the interactions with calix[4]arene glycomimetics at the cell level was investigated. An aggregation test was used to establish the efficacy of these ligands. Great variations in the responses were observed. Glycomimetics directed against LecB yielded the highest numbers of aggregates for strains from all clades. The use of a PAO1Δ*lecB* strain confirmed a role of LecB in this aggregation phenotype. Fucosylated calix[4]arene showed the greatest potential for a use in the prevention of *P. aeruginosa* infections.

## Introduction

Opportunistic infections are of major concern around the world. They can lead to community or nosocomial infections among individuals showing weaknesses in their protective barriers. *Pseudomonas aeruginosa* is one of the main human opportunistic pathogens, and can be found in multiple outdoor habitats leading to human exposures. It can be the etiological agent of several infections such as folliculitis, keratitis, otitis but also pneumopathies in cystic fibrosis patients which can evolve into severe septicemia and death (Forkner et al., 1958). *P. aeruginosa* is divided into multiple lineages but has a panmictic organization (Kidd et al., 2012; Dettman et al., 2015). *P. aeruginosa* sequenced genomes showing the most significant differences with the classical laboratory strains such as PAO1 and PA14 are those of the PA7 clade (Boukerb et al., 2015; Freschi et al., 2015). This lineage was initially defined as a taxonomic outlier (Roy et al., 2010; Valot et al., 2015), and has diverged early from the other lineages (Gomila et al., 2015; Hilker et al., 2015). The multidrug-resistant PA7 strain was isolated from a patient in Argentina, and found to harbor several pathogenicity islands and putative virulence factors (Roy et al., 2010).

Among its key properties involved in host colonization, *P. aeruginosa* has selected a “glyco-strategy” favoring oligosaccharide-mediated recognition and adhesion to host cells. This strategy involves carbohydrate binding proteins such as lectins and other adhesins like fimbrial proteins and flagella. The lectin-carbohydrate interactions are characterized by their high specificity and multivalency to generate higher affinity of binding. Two soluble lectins, LecA and LecB, involved in adherence and biofilm formation have been described in *P. aeruginosa*. These lectins act as virulence factors through their carbohydrate binding ability. They promote the adhesion of *P. aeruginosa* to epithelial cells and cause alveolar damages (Chemani et al., 2009; Boukerb et al., 2014). They also inhibit ciliary beating of epithelial cells (Mewe et al., 2005). LecA and LecB bind to galactose and fucose, respectively (Gilboa-Garber, 1982), and their glycan-binding spectra cover a wide range of antigens (ABH, Lewis, P and I systems) present on human tissues or bacterial cell walls (Imberty et al., 2004). Glycomimetics have been synthesized to prevent lectin-dependent adhesion to host cells. Calixarene scaffolds decorated with galactose and fucose were found particularly efficient (Bernardi et al., 2013; Cecioni et al., 2015). Such molecules were found to increase *P. aeruginosa* aggregation and to reduce damages of alveolar tissues (Boukerb et al., 2014).

However, considering the many environmental matrices and living hosts that can be colonized by *P. aeruginosa*, one could hypothesize strong selective pressures on lectins that could lead to modified sugar affinities. Such changes could prevent therapeutic uses of some glycomimetics. In order to identify such events, genetic diversity analyses of *lecA* and *lecB* from a large panel of environmental and clinical *P. aeruginosa* strains were conducted. The relation between the observed *lec* types and sugar-based calixarene glyco-clusters induced aggregations was investigated. Most significant modifications at the *lec* loci were observed in the PA7 clade. This clade was thus used as a reference to investigate genomic instability around these loci. Three PA7-related genomes were sequenced and annotated for this work (Boukerb et al., 2015). The crystal structure of LecB_PA7_ was resolved to investigate the incidence of some amino acids changes on the structural organization of the LecB tetramers and sugar binding site. This work showed genomic and genetic changes at the *lec* loci indicative of ongoing adaptive processes among *P. aeruginosa* which can lead to their loss or changes in the sugar ligand affinities of their encoded lectins.

## Materials and methods

### Bacterial strains, growth conditions, and DNA extractions

A total of 148 *P. aeruginosa* strains were used in this study (Supplementary Tables S1, S2). Environmental strains (*n* = 74) (Lavenir et al., 2007, 2014; Petit et al., 2013) belonging to *P. aeruginosa* were obtained from the EML-BRC (Environmental Microbiology Lyon—Biological Resource Center) collection (http://eml-brc.org) of the French Network of Biological Resource Centers (FBRCMi; www.fbrcmi.fr). Clinical strains (*n* = 70) coming from non-CF infections (*n* = 40; recovered from cases of otitis, urinary tract, and wound infections) were provided by the French “Collège de Bactériologie, de Virologie et d'Hygiène des Hôpitaux” (Paris, France). Isolates from CF patients (*n* = 30) were also included, and previously reported in (Doléans-Jordheim et al., 2009). Reference strains, PAO1 (Holloway, 1955) and PA7 (Roy et al., 2010) and isogenic mutants PAO1Δ*lecA* and PAO1Δ*lecB* (Boukerb et al., 2014) were used to validate the PCR screenings and aggregation assays. *P. aeruginosa* total DNA was extracted and visualized according to Johnson et al. (1994).

### PCR screenings and genetic diversity analyses

MLST analyses were performed according to Curran et al. (2004). PCR primers used to detect *lecA* and *lecB* among the *P. aeruginosa* collection were designed using conserved DNA regions among PAO1, PA14, PA7, and LESB58 genomes (Supplementary Table S3). These *lecA* and *lecB* PCR screenings were validated on PAO1 and PA14 DNA extracts. PCR reactions were performed in 25 μL using a *Taq* DNA polymerase from Invitrogen (Cergy-Pontoise, France) by following the manufacturer's instructions. Annealing temperatures are indicated in Supplementary Table S3. Each PCR test was, at least, duplicated, and when needed, bacterial colony blots were analyzed to validate the presence or absence of targeted genes. Colony blots DNA hybridizations were performed using radioactively labeled (^32^P) *lecA* and *lecB* PCR products from strain PAO1 as described previously (Pallud et al., 2001). PCR products were visualized after electrophoresis using a 2% agarose gel, staining with ethidium bromide (at 5 mg/L for 15 min) and UV light exposure. PCR products were sequenced by the Biofidal (Villeurbanne, France) sequencing platform. Their DNA sequences were analyzed using the BioEdit software. Similarity and identity searches of the DNA/amino acid sequences were performed using the NCBI website (http://www.ncbi.nlm.nih.gov/).

Multiple alignments of DNA and protein sequences were computed using CLUSTALX v2.1. These alignments were visualized using Seaview (Gouy et al., 2010). BLAST analyses were run at NCBI and insertion sequences were characterized using the IS biotoul website (http://www-is.biotoul.fr/). The phylogenetic relationships between DNA or amino acid sequences were computed by distance matrix methods according to Galtier et al. (1996). Neighbor-joining trees and 1000 bootstrap replicates were generated. The adjusted Wallace coefficients enabling comparisons of partitions between genetic markers (*lec* and MLST types) were computed according to Severiano et al. (2011) using UMMI web site at Universidade de Lisboa (http://www.comparingpartitions.info/). Homoplasy test was performed using Splitstree4 (Huson and Bryant, 2006). The DNA and amino acid sequences generated in this work have been assigned GenBank accession numbers KT182468 to KT182473.

### Comparative genomics

Assembled genomes of three PA7-related strains, i.e., EML528, EML545 and EML548 used in this study have been deposited at DDBJ/EMBL/GenBank under the following accession numbers: LFXS00000000.1 for EML528, LGJE00000000.1 for EML545, and LFXR00000000.1 for EML548 (Boukerb et al., 2015). These genomes were aligned against the PAO1 and PA7 genome sequences using Mauve (v2.3.1; http://asap.genetics.wisc.edu/software/mauve/). The Magnifying Genome platform (MaGe, http://www.genoscope.fr/agc/mage/) was used to compare CDS contents and analyze some other features (Vallenet et al., 2013). Comparative analyses of *lecA* and *lecB* genomic regions among PAO1, PA7, and related strains were performed using ACT (Artemis Comparison Tool) at http://www.webact.org. RGP (regions of genomic plasticity) among these regions were detected by looking for synteny breaks due to an integration of DNA pieces above 900 bp. Small indels of a few nucleotides were not considered and not observed among the *lec* loci.

### *P. aeruginosa* PA7 *lecB* sub-cloning, over-expression, and glycan specificity tests

Cloning of the *lecB*_PA7_ gene into pET25b vector (Novagen) was carried out by a PCR strategy using primers described in Supplementary Table S3. LecB_*PA*7_ over-expression was performed in *Escherichia coli* BL21 (DE3) (Novagen). Cells were grown in LB (Luria-Bertani broth, Sigma-Aldrich, USA) under orbital shaking (200 rpm) at 37°C until reaching an OD_600_ (optical density at 600 nm) of 0.6 prior induction with 0.5 mM isopropylthiogalactoside (IPTG) for 3 h at 30°C. Cell lysates were applied onto a D-mannose-agarose column (Sigma-Aldrich, USA) which was equilibrated with a buffer (20 mM TrisHCl pH 7.5, 100 mM NaCl, 100 μM CaCl_2_). After washing out unbound proteins, LecB_PA7_ was eluted using the same buffer but supplemented with 300 mM D-mannose. The purified protein was dialyzed for 1 week at 4°C.

Purified recombinant LecB_PA7_ was labeled with Alexa Fluor 488 (Invitrogen, France) according to the manufacturer's instructions and repurified on a D-Salt polyacrylamide desalting column (Pierce, France). Alexa-labeled LecB_PA7_ was used for glycan array screening with the standard procedure of the Protein-Glycan Interaction Core (H) of the Consortium for Functional Glycomics, USA. The Glycan Array version 5.2 that contains 609 glycans was screened.

### Microcalorimetry

Recombinant LecB_PA7_ was dissolved in a 20 mM TrisHCl, pH 7.5, NaCl 150 mM, 100 μM CaCl_2_ buffer. Carbohydrate ligands were dissolved in the same buffer, degassed, and loaded in the injection syringe. ITC was performed using ITC200 microcalorimeter (Malvern Instruments, UK). The LecB_PA7_ solution was placed in a 200 μL sample cell at 25°C. Titration was performed with 20 injections of 2 μL carbohydrate ligands every 120 s. The experimental data were fitted on a theoretical titration curve using the “Origin” software (RITME Informatique, France), with Δ*H* (enthalpy change), K_a_ (association constant) and n (number of binding sites per monomer) as adjustable parameters. Free energy change (ΔG) and entropy contributions (TΔ*S*) were derived from the equation Δ*G* = Δ*H* − TΔ*S* = −RT ln K_a_ (with T as the absolute temperature and *R* = 8.314 J mol^−1^ K^−1^). Two independent titrations were performed for each tested ligand.

### Protein crystallography

Crystals of LecB_PA7_ complexed with Lewis^a^ tetrasaccharide (Elicityl, Crolles, France) were obtained by the hanging drop vapor diffusion method using 2 μL of drops containing a 50:50 (v/v) mix of protein and reservoir solution at 19°C. The protein at 10 mg/mL in 20 mM HEPES pH 7.5 and 1 μM CaCl_2_ was incubated with the ligand at 10 mM during 1 h at room temperature prior to co-crystallization. Crystal plates were obtained in 2 days from solution containing 14% PEG6000, 0.2 M lithium chloride, and 0.1 M sodium acetate pH 4.6. Plates were transferred in a solution where PEG6000 concentration was increased to 26% for cryoprotection prior mounting in a cryoloop and flash-freezing in liquid nitrogen. Diffraction data were collected at 100 K at the European Synchrotron Radiation Facility (Grenoble, France) on beamline BM30A using a ADSC Q315 CCD detector. The data were processed using iMosflm (Battye et al., 2011). All further computing was performed using the CCP4 suite (Winn et al., 2011). Data quality statistics are summarized in Supplementary Table S4. The structure was solved by molecular replacement using PHASER and the monomer coordinates of 1GZT as search model, according to McCoy et al. (2007). Five percent of the observations were set aside for cross-validation analysis, and hydrogen atoms were added in their riding positions and used for geometry and structure-factor calculations. The structure was refined using restrained maximum likelihood refinement in REFMAC 5.8 (Murshudov et al., 2011) iterated with manual rebuilding in Coot (Emsley et al., 2010). Incorporation of the ligand was performed after inspection of the 2Fo-DFc weighted maps. Water molecules, introduced automatically using Coot, were inspected manually. The stereochemical quality of the models was assessed with the program Molprobity (Chen et al., 2010), and coordinates were deposited in the Protein Data Bank under code 4UT5.

### Bacterial aggregation assays through imaging flow cytometry analysis

Aggregation assays were performed according to Boukerb et al. (2014) but aggregates were visualized and analyzed by Amnis FlowSight® cytometry. Image acquisitions and analyses were performed with the ImageStream system (Amnis Corporation, Seattle, WA, USA) and the ImageStream Data Exploration and Analysis Software (IDEAS, Amnis). *P. aeruginosa* cells were exposed to 100 μM glucosylated, mannosylated, galactosylated, and fucosylated glycoclusters calix[4]arene ligands obtained from Boukerb et al. (2014) (Supplementary Figure S1). Results were compared with those obtained from PBS and monovalent ligands. For each condition, the aggregation properties were analyzed over 20,000 cellular events. Kruskal–Wallis rank sum tests were used to determine conditions favoring the formation of the highest number of bacterial aggregates. *P* < 0.05 were considered statistically significant. These tests were computed using the R (R Development Core Team, 2015) software.

## Results

### Prevalence and diversity of *lecA* and *lecB*

**Figure 1:**
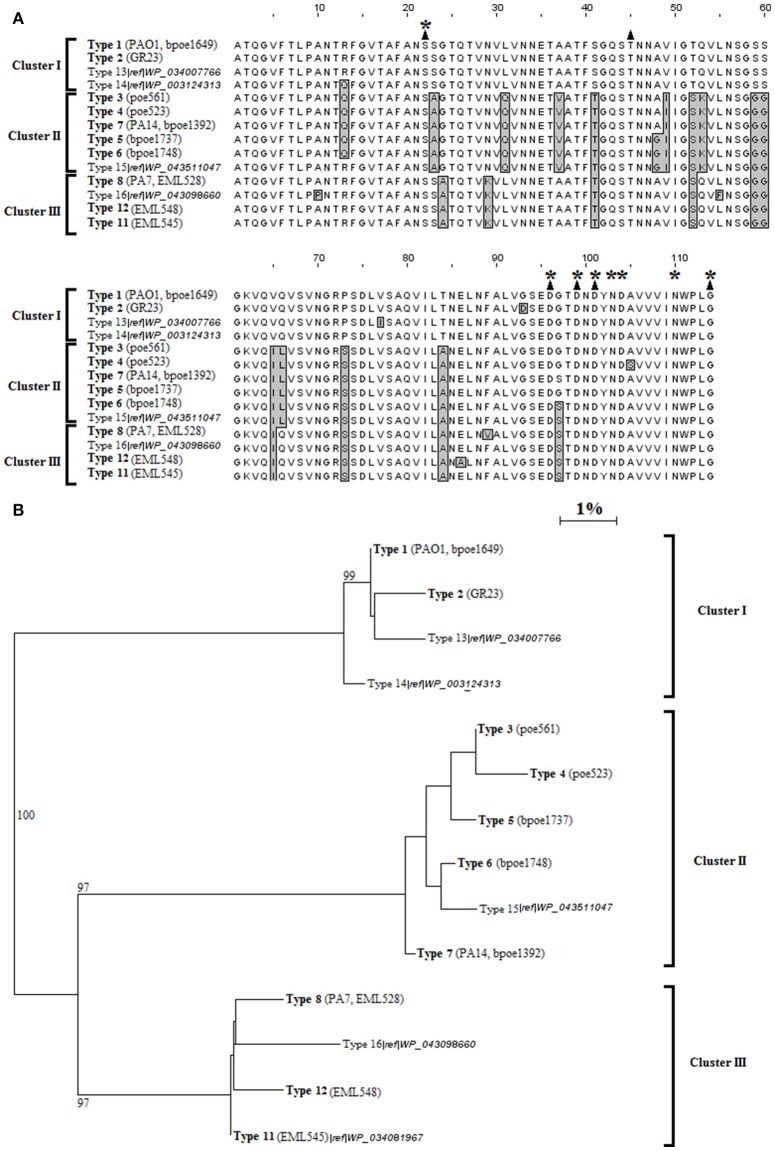
**LecB types among *P. aeruginosa*. (A)** Alignment of the 14 types of LecB amino acid sequences detected in this work (bold text) or recovered from the GenBank database (faint text). Amino acids different from those of the PAO1 sequence (total of 25 positions) are boxed and in gray. Black triangles indicate the amino acids participating in sugar binding, and asterisks the amino acids participating in calcium coordination. **(B)** Neighbor-Joining phylogenetic tree of the 14 LecB sequence types. One hundred and fourteen sites were analyzed. Horizontal lines represent the divergence % between pairs of sequences. Bootstrap values are indicated on branches. Representative strains of the 12 types identified in this work are indicated. Types 9 and 10 are not indicated but showed, respectively, a single change with type 1, and a single one with type 6.

*lecA* and *lecB* databases were built by retrieving the *P. aeruginosa* sequences available at GenBank and at the *Pseudomonas* Genome database (Winsor et al., 2015) and by performing *lecA* and *lecB* PCR screenings over a collection of environmental and clinical strains (Figure 1 and Supplementary Figure S2). A total of 292 PCR products from 147 strains were sequenced (Supplementary Tables S1, S2). DNA blot analyses were performed to verify PCR false-negative results (data not shown). Most strains showed PCR products of the expected size. Two strains (poe1196 and poe1293) yielded *lecA* PCR products of about 2 kb, and were found harboring an IS*Psp4*-like element which is part of the IS*30* family (Supplementary Table S1). This IS was inserted at *lecA* nucleotide position 288 (according to PA2570 numbering). In addition, the multi-drug resistant PA7 strain and strains EML528, EML545, and EML548 were found lacking *lecA*. These latter strains were found to be part of the PA7 clade (see below).

These data sets allowed the identification of genetic variants that were classified into Lec types according to their amino acid sequences (Supplementary Figure S3). Low divergence was observed between LecA types (<3%). More divergences were observed between LecB ones (<16%). LecA was divided into 17 types (Supplementary Figure S2), and LecB into 14 ones (Figure 1). It is noteworthy that the LecA typings did not perfectly match phylogenetic groupings inferred from MLST DNA sequences (Supplementary Figure S4). Adjusted Wallace coefficient analysis showed that ST defined by MLST will have a same LecA type for 84% of the strains while this probability was of 98% for LecB. LecA types 2 and 4 were found restricted to the PAO1 MLST group but types 1 and 3 were distributed among both the PAO1 and PA14 groups (Supplementary Figures S3, S4). However, the phi test did not find statistically significant evidence for recombination at this locus among *P. aeruginosa* (*p* = 0.23). These changes are thus likely to have occurred independently. Three significant LecB clusters could be inferred by these analyses (Figure 1). LecB type 1 (67%) and 2 (1%) were strictly found in the PAO1 phylogenetic cluster (Supplementary Figure S4). The PA7 and alike strains (EML528, EML545, and EML548) were grouped in a same phylogenetic cluster that was found strictly harboring LecB cluster III sequences (types 8, 11, and 12; Figure 1). LecB cluster II sequences, mainly shared by STs of the PA14 phylogenetic group, showed signs of genomic instability, with types 5 and 6 being also recorded in the PAO1 group (Supplementary Figure S4). Their respective pattern of distribution suggested horizontal transfers between the PA14 and PAO1 sub-clades. The phi test was in agreement with this observation, and found statistically significant evidence for recombination events among the *P. aeruginosa* collection between the *lecB* loci (*p* = 0.009). A total of 16 LecA and LecB combinations were observed (Supplementary Figure S4). Nine LecA/LecB combinations were observed in the PAO1 cluster with the dominant 1/1 combination at 61%. Four LecA/LecB combinations were observed in the PA14 cluster, and three combinations in the PA7 one.

### Inferred incidence of amino acid changes on lectin structures

**Figure 2:**
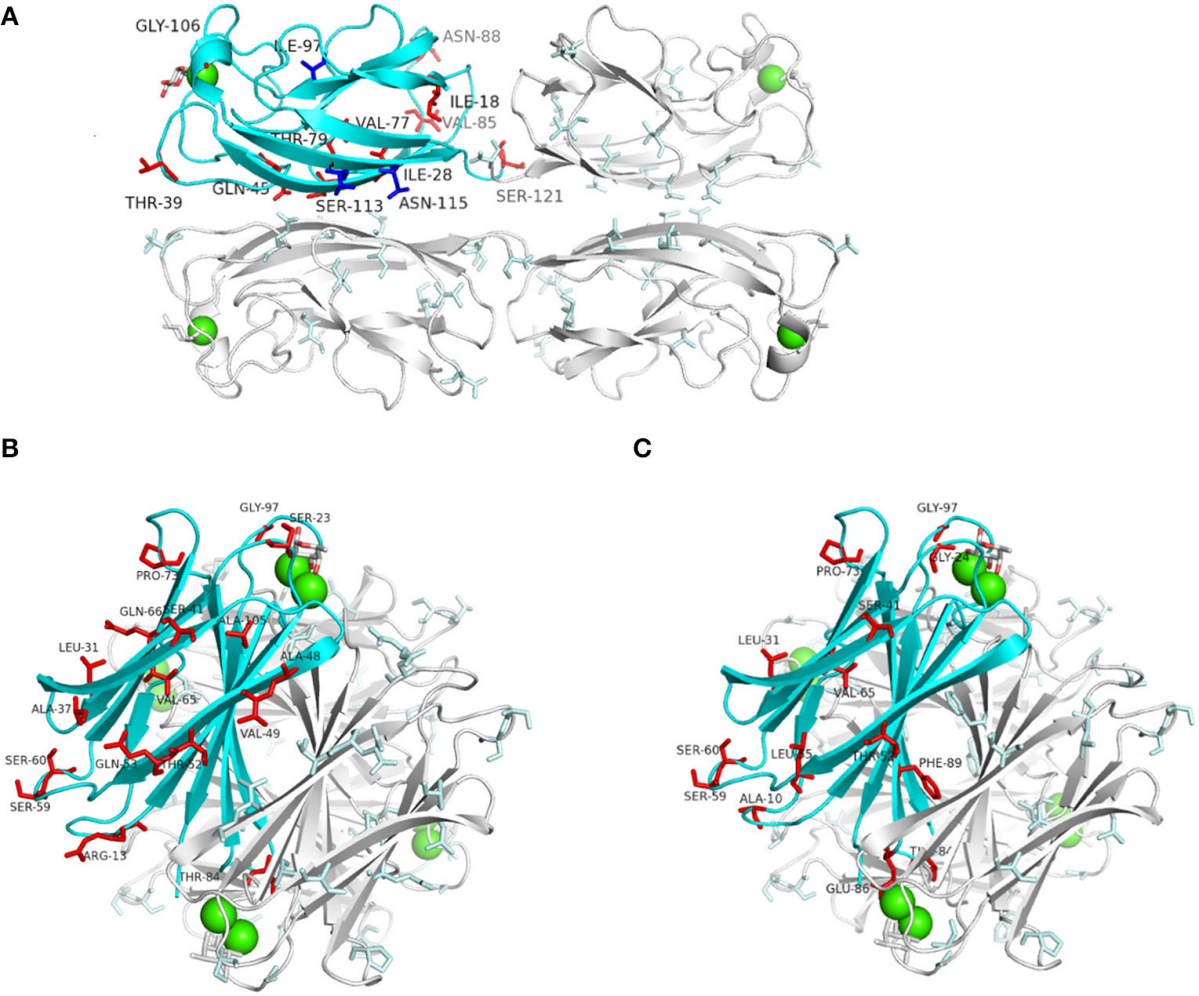
**Predicted incidence of amino acids changes on LecA_*PAO1*_ and LecB_*PAO1*_ crystal structures co-crystallized with galactose (LecA: code 1OKO) and fucose (LecB: code 1GZT)**. One protein monomer in each structure of LecA and LecB is represented by a blue ribbon. Calcium ions are represented as green spheres, and monosaccharides as sticks. **(A)** LecA_PAO1_ structure with all amino acids change observed in lineage I (red sticks) and lineage II (blue sticks; Supplementary Figure S1). **(B)** LecB_PAO1_structure showing the incidence of the observed amino acids changes among cluster II sequences (Figure 1). **(C)** LecB_PAO1_ structure showing the amino acids changes of cluster III sequences (Figure 1). All structural figures were drawn with PyMOL Molecular Graphics System.

The amino acid variations of LecA and LecB were mapped on the crystal structures of lectins derived from PAO1 (Mitchell et al., 2002, 2005; Cioci et al., 2003). Amino acid changes observed among LecA lineage I (16% of the tested strains) are located on loops that are not directly involved in structure stabilization or ligand binding (Figure 2A). However, LecA type 5 showed an Asn115Ser change (Asparagine to Serine at position 115) that can prevent a hydrophobic contact with Phe82 on the neighboring monomer, therefore destabilizing the LecA tetramer. LecB cluster I observed changes do not have any functional consequences. However, LecB cluster II variants showed Val49Ile and Thr52Ser changes that can affect the stability of the LecB tetramer (Figure 2B), and a Ser23Ala modification demonstrated to double the association constant for fucose (Adam et al., 2007). LecB type 6 and type 7 showed a Gly97Ser close to the fucose-binding site, affecting the binding of fucosylated oligosaccharides. LecB type 8 of cluster III showed a Phe89Val which is involved in dimer formation (Figure 2C), and Gly97Ser and Gly24Ala changes in the binding site area. Other LecB cluster III amino acid changes should not impact the tetramers. LecB_PA7_ being clearly impacted on key residues while comparing with LecB_PAO1_, its affinities for various sugar ligands were investigated.

### Comparison of affinities between lecB_PA7_ and lecB_PAO1_

**Figure 3:**
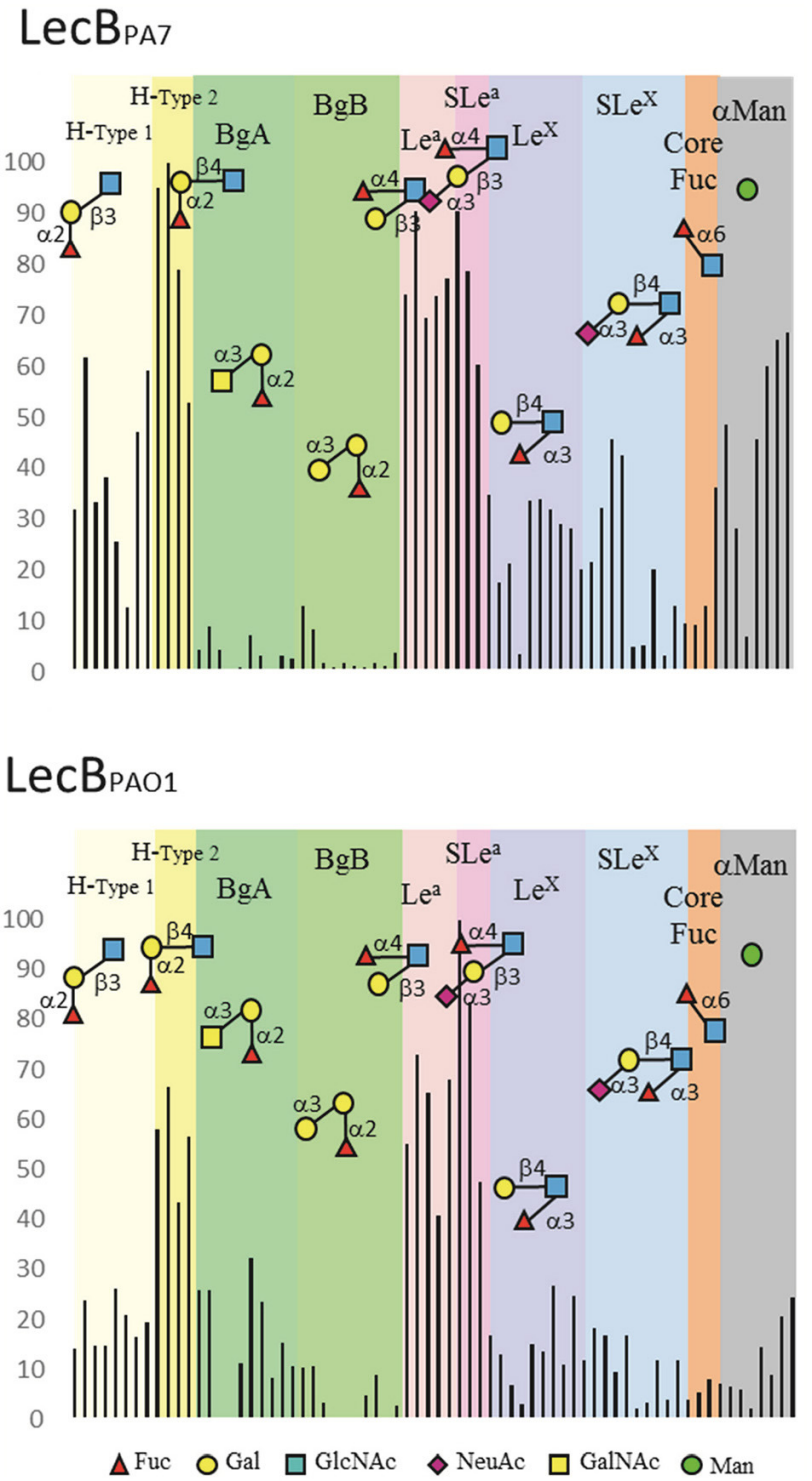
**Selected datasets from the glycan array analysis performed on PA7 and PAO1 LecB forms**. Binding is represented as relative fluorescent intensities (y-axis) obtained with LecB_PA7_ (at 200 μg/mL) and LecB_PAO1_ (at 10 μg/mL) labeled with Alexa 488. A value of 100% is representing the response (RU) for the oligosaccharide with the highest affinity. Oligosaccharides (x-axis) have been grouped using a color code matching blood group families, and a representative molecule was displayed schematically. Yellow: blood group O (sub-type H1 and sub-type H2); Green: Blood group A and Blood group B, Pink: Lewis a and sialyl-Lewis a; Blue: Lewis x and sialyl-Lewis x; Orange: core fucose on N-glycans; Gray: any terminal α-mannose.

Purified LecB_PA7_ produced in *E. coli* were assayed on the Glycan Array v5.2 from the Consortium for Functional Glycomics (*n* = 609 glycans). Only glycans with terminal fucose or mannose residues were recognized by the lectin. Stronger signals were obtained for biantennary glycans that present fucosylated epitopes at the top of two long chains, then to multiple branched glycans with several fucose residues such as Lewis^y^ epitope (Supplementary Table S6). Data previously obtained for LecB_PAO1_ (on 465 glycans, available on CFG web site) on epitopes containing only one terminal fucose or mannose residue were extracted and compared with those of LecB_PA7_ (Figure 3 and Supplementary Figure S5). LecB_PA7_ recognized more efficiently fucose when present on position 2 of galactose as in blood group O/H, and on position 3 and 4 of *N*-acetylglucosamine (GlcNAc) as in Lewis^x^ (Le^x^) and Lewis^a^ (Le^a^) epitopes. Comparisons with LecB_PAO1_ showed H-type 2 (αFuc1-2βGal1-4GlcNAc) to be preferred by LecB_PA7_ rather than H-type 1 (αFuc1-2βGal1-3GlcNAc), and Lewis^a^ (αFuc1-4[βGal1-3]GlcNAc) rather than Lewis^x^ (αFuc1-3[βGal1-4]GlcNAc). However, a substitution by a Gal or a GalNAc on H-disaccharide was not tolerated; so, blood group A and B were better recognized by LecB_PAO1_ rather than LecB_PA7_. On the opposite, sialic acid substitution on Lewis^a^ was well tolerated, and sialyl Lewis^a^ (sLe^a^) bound as efficiently as Lewis^a^. LecB_PA7_ binds more strongly to mannose than LecB_PAO1_, and appears less specific for fucose.

Affinity data sets were recorded for the interaction of LecB_PA7_ with monosaccharides and oligosaccharides using titration microcalorimetry (Supplementary Figure S6 and Supplementary Table S5) and compared with data previously obtained with LecB_PAO1_ (Perret et al., 2005; Sabin et al., 2006). The methyl glycosides (methyl α-l-fucoside: FucOMe and methyl α-d-mannoside: ManOMe) were used in order to block the anomeric position and avoid mutarotation in solution for the native fucose and mannose. LecB_PA7_ demonstrated a stronger affinity for FucOMe (*K*_d_ = 2.2 μM) than for ManOMe (*K*_d_ = 73 μM). Affinity for the Lewis^a^ oligosaccharide (*K*_d_ = 2.01 μM) was in the same range as for fucoside. Comparison with LecB_PAO1_ (Supplementary Table S5) indicates that both lectins have the same affinity for mannoside, and same thermodynamic contribution to the free energy of binding with slightly favorable entropy of binding. However, LecB_PAO1_ bound FucOMe and Lewis^a^ more efficiently than LecB_PA7_, with factor 5 and 10 respectively. This confirms that LecB_PA7_ is less specific for fucosylated oligosaccharides and can likely bind to other glycans.

### Structural basis for the differences between lecB_PA7_ and lecB_PAO1_

**Figure 4:**
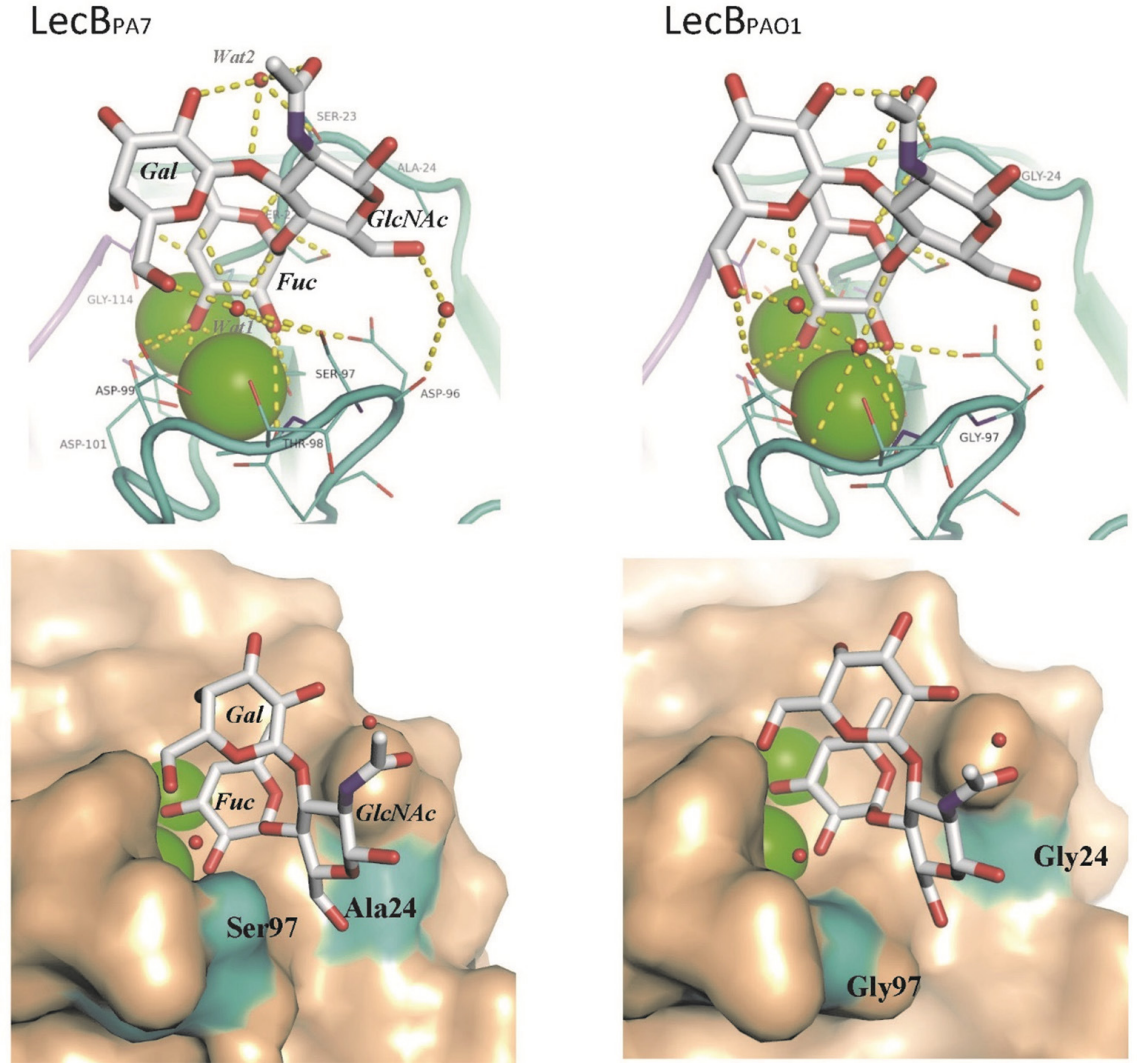
**Comparison of the crystal structures of LecB_*PA7*_ (this work) and LecB_*PAO1*_ (PDB code 1W8H) complexed with Lewis^a^**. Calcium ions are depicted as green spheres and water molecules as red spheres. Top Panel: detail of the binding sites with hydrogen bonds as dotted yellow lines. Bottom Panel: surface representation of the binding site. Two amino acids with a modified positioning have been colored in cyan.

One crystal of LecB_PA7_ complexed with Lewis^a^ tetrasaccharide diffracted to 1.75 Å resolution (Supplementary Table S4) and the structure could be solved in the P2_1_ space group, with one tetramer per asymmetric unit. All of 114 amino acids could be located in the electron density, as well as the 8 calcium ions (2 per sites). The whole tetrasaccharide was clearly visible in three sites, and only one reducing glucose was disordered in monomer D (Supplementary Figure S7). The structure of the LecB_PA7_/Lewis^a^ complex was compared to the one previously obtained for LecB_PAO1_/Lewis^a^ complex (Perret et al., 2005). Both structures share the same overall organization with rmsd (root-mean-square deviation) of 0.4 Å over the 456 amino acids of the tetramer. When looking at the binding site, the fucose residue adopts the same orientation in both lectins. No amino acids change involved in calcium coordination or in the hydrogen bonding to fucose was recorded (Figures 2B,C). The other two sugar residues of Lewis^a^, i.e., galactose and GlcNAc, present a slight shift in response to the amino acids changes and the modified network of bridging water molecules (Figure 4). In LecB_PA7_, Ser97 (that replaces Gly97 in LecB_PAO1_) establishes a hydrogen bond with one of the two structural water molecules that bridges between the glycan ligand and the protein surface. The water molecule (*Wat1* in Figure 4) is moved away from the protein backbone. As a result, the water molecule network is disturbed in this area, which can correlate with the stronger entropy barrier measured for LecB_PA7_ when binding to sugars. The other variation, i.e., Ala24Gly results in an additional hydrophobic contact with the methyl group of Ala24 close to the CH_2_ group at C6 of the GlcNAc residue. As displayed in Figure 4, the two mutations can therefore play a role on the fucose affinity by disturbing the water network and on the Lewis^a^ affinity by modifying the shape of the binding site on the surface.

### Genome-wide analyses of lecB cluster III strains

**Figure 5:**
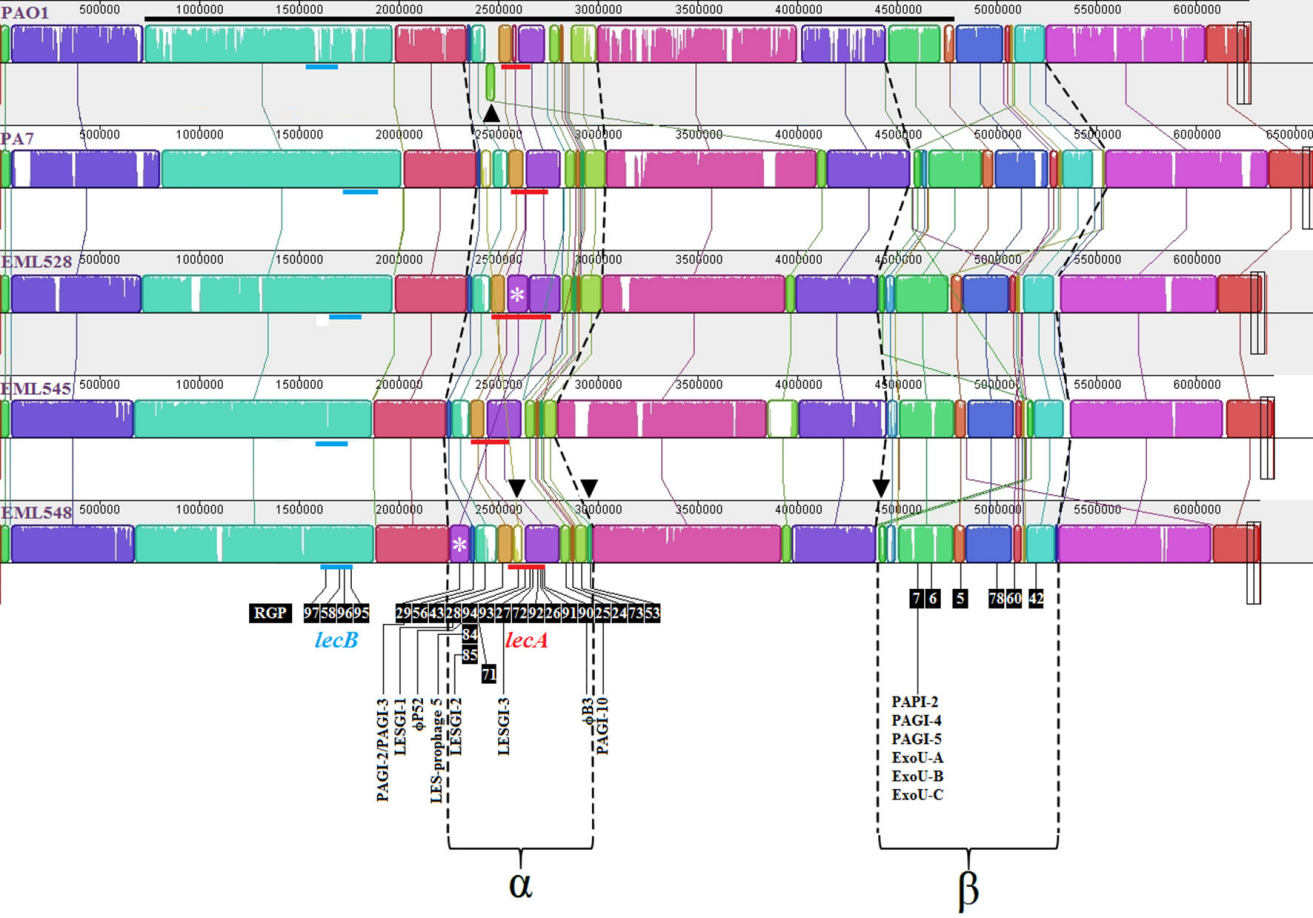
**MAUVE comparisons of *P. aeruginosa* PAO1, PA7, EML528, EML545, and EML548 genomic organizations**. DNA segment going from 727,314 to 4,788,531 of the PAO1 genome was manually inverted for a better match of syntenic groups (long black line). Colored blocks outline linked genomic regions between strains (locally collinear blocks named LCB). White regions correspond to sequences that are not aligned and probably contain sequence elements specific of a particular genome. The height of the colored profile within each LCB indicates the average degree of sequence conservation between the aligned regions. Horizontal red lines represent the *lecA* genomic regions localized in the analyzed genomes with inferred RGP (according to Figure 6). Horizontal blue lines represent the *lecB* genomic regions localized in the analyzed genomes with inferred RGP (numbers are those of the literature or from Supplementary Figure S8). α and β indicate two large regions of genomic instability. Asterisks represent PAGI-2 element identified in EML528 and EML548. The green block below the center line in PAO1 indicates a reverse complement (inverse) orientation (see arrowhead of Top Panel). Arrowheads of the Last Panel highlight major rearranged genomic blocks.

**Figure 6:**
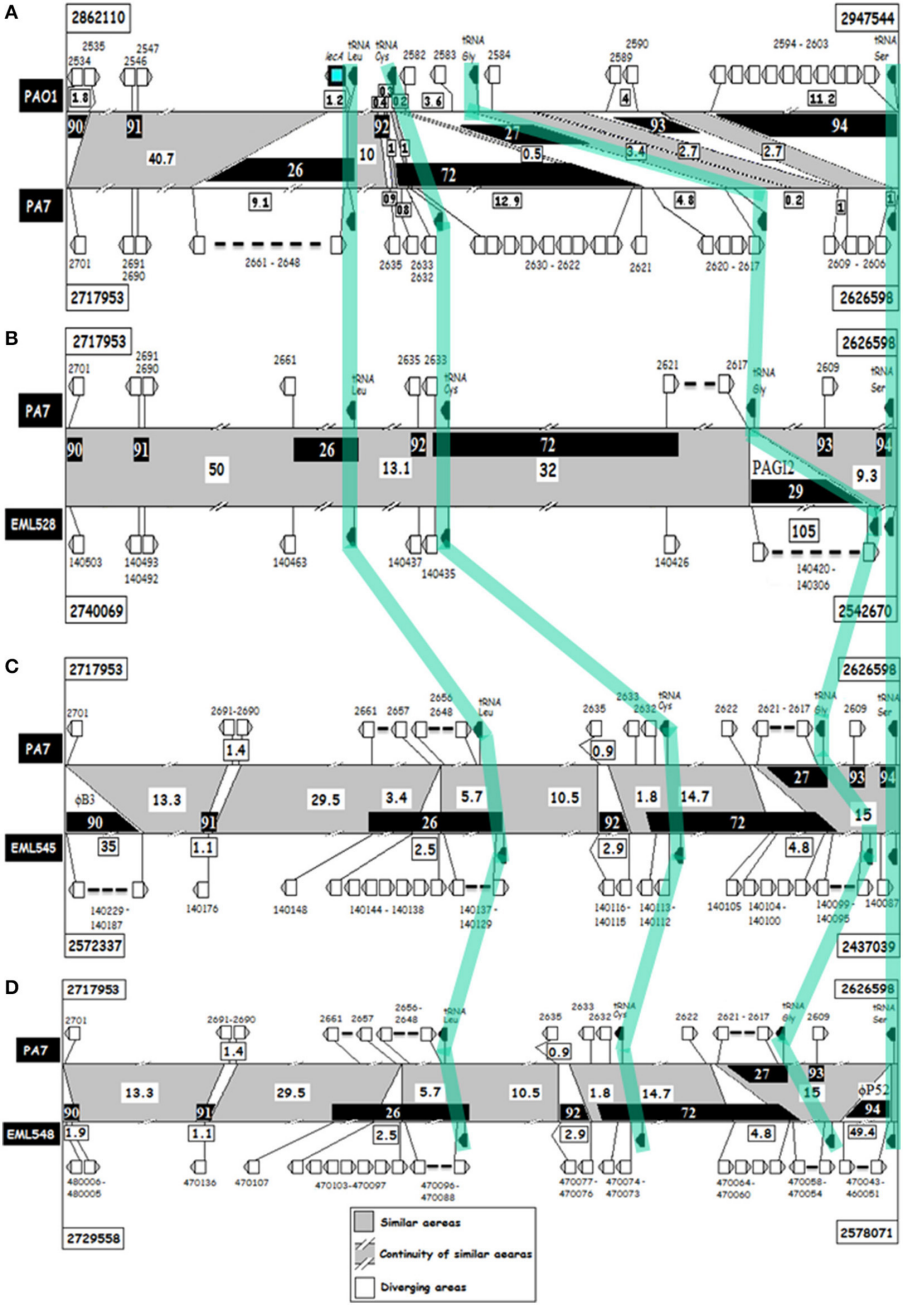
**ACT analyses of *lecA* genomic regions of *P. aeruginosa* strains PAO1, PA7, EML528, EML545 and EML548. (A)** PAO1 (top) against PA7 (bottom); **(B–D)**, PA7 (top) against, respectively, EML528 **(B)**, EML545 **(C)**, and EML548 **(D)** regions (bottom). Horizontal black lines indicate the genome sequences compared over a length of about 80 kb. Gray indicates conserved regions, and white the variable ones. Distances between variable regions are given in kb. Regions of genomic plasticity (RGP) are indicated in black-boxes. Selected CDS (above or below the ACT panels), and tRNA genes (in black) are shown. Orthologous tRNA genes between pairs of genomes are highlighted by a light-green line crossing the ACT panels.

Assembled draft genomes of three PA7-related strains (EML528, EML545, and EML548) were generated (Boukerb et al., 2015) in order to investigate the LecB cluster III strains showing major changes at their *lecA* (deleted) and *lecB* genomic regions. MAUVE comparisons were performed (Figure 5). Two large regions of genomic instability named α (2,390,740–3,038,628) and β (4,569,960–5,542,308) were detected. One of these regions was found to harbor RGP26 (Mathee et al., 2008) which is containing *lecA* among PAO1. Among the α region, at least 14 RGP defined in other studies were recorded (e.g., Mathee et al., 2008; Roy et al., 2010; and Klockgether et al., 2011). Five novel RGP were identified and named RGP90, RGP91, RGP92, RGP93, and RGP94 (Figures 5, 6). Some of these RGP matched DNA segments smaller than 5 kb but were confirmed, in some instances, to be targets for longer genetic elements e.g., RGP90. The *lecB* loci genomic environments were not subjected to similar large scale rearrangements but rather to small ones (up to 16 kb).

The α genomic region harboring *lecA* showed: (1) an inverted and distantly positioned DNA region between the PAO1-UW (3,028,630–3,077,236) and PA7 (4,096,626–4,144,187) and alike genomes (top arrowhead on Figure 5) including an additional 116 kb DNA segment among the EML545 genome that did not match other sequences in the databases; (2) RGP29 (see asterisk on Figure 5) in EML528 harboring the PAGI-2 genomic island (Supplementary Figure S8A); PAGI-2 was also observed in EML548 but at a distinct genomic position (2,254,558–2,359,617; Supplementary Figure S8B); (3) RGP94 shown to be colonized by a DNA segment of 49.4 kb in EML548 which was located in a different position in PA7 (with 22% of sequence cover and 84% of sequence identity; see bottom left arrowhead on Figure 5), and (4) an unstable region (dark green block, bottom middle arrowhead of Figure 5) of 19.1 kb absent in PAO1 and positioned differently among PA7 and alike genomes. The β variable region was found to match the area involved in the major DNA inversion observed among PAO1 strains, with the localization of RGP5-7, RGP42, and RGP60 (Mathee et al., 2008), and RGP78 (Roy et al., 2010). A large indel was observed in this area (green block, bottom right arrowhead; Figure 5). This block was shared between PA7 (4,584,072–4,620,550; 76% of sequence cover and 96% of sequence identity) and closely related genomes, but at a different position in EML545. It was found to match pUM505, a conjugative plasmid described by Ramirez-Diaz et al. (2011) which is encoding a chromate resistance determinant.

Around 80 kb surrounding *lecA* (or RGP26 in PA7 and related genomes) was subjected to deeper investigations of DNA signatures indicative of genomic plasticity such as integrase CDS and breaks in syntheny. The borders and size of the DNA segments found to match large and small genomic indels (insertions/deletions) are presented in Supplementary Table S7. These were often found encoding phage-like proteins, integrases, transcriptional regulators, and various enzymes, factors and transporters. Interestingly, the *lecA* area was found to contain several tRNA gene sequences (tRNA^Leu^, tRNA^Cys^, tRNA^Gly^, and tRNA^Ser^), and these are often targeted by integrative elements. The *lecA* CDS was in frame and proximal to a tRNA^Leu^ gene. This zone was previously defined as RGP26 in PA7 (Supplementary Table S7). This RGP was shown to contain CDS indicative of a likely integration of phage DNA which could have led to the observed *lecA* deletion in the PA7 clade. RGP90 and RGP94 were also found colonized by phage DNA, suggesting the *lecA* area to be frequently challenged by phage attacks. In fact, a 35 kb-long DNA segment was found at RGP90 and matched a bacteriophage B3 element (Figure 6 and Supplementary Figure S9). RGP94 is bordered by a tRNA^Ser^ gene which appeared to have been targeted by a P52 bacteriophage in the EML548 genome (Supplementary Figure S10). It is noteworthy that the tRNA^Cys^ gene was found in a region of great instability harboring RGP92 which harbored a CDS encoding a fimbrial protein and a transcriptional protein of the MerR family in EML545 and EML548 genomes (2.9 kb).

Comparative analysis of *lecB* genomic regions in *P*. *aeruginosa* PAO1, PA7, and the EML strains revealed a higher conservation than the *lecA* ones. Only four RGP could be detected (Supplementary Figure S11 and Supplementary Table S8). The *lecB* gene was physically linked to RGP96. This RGP was delimited by the stop codon of *lecB* in PAO1, and PA7 and closely related genomes. Small insertions of 0.6 kb in PAO1 and 0.9 kb in PA7 and alike genomes were detected at this RGP but these inserts were unrelated. These changes in the terminator region could be affecting the *lecB* expression levels but the promoter region did not appear to vary from one strain to another. A tRNA^Arg^ gene favored the integration of foreign DNA at RGP58. No phage DNA, integrase CDS or adhesin-like CDS could be detected in the analyzed *lecB* area.

### *P. aeruginosa* cells aggregation behavior according to lecA/lecB types combinations

**Figure 7:**
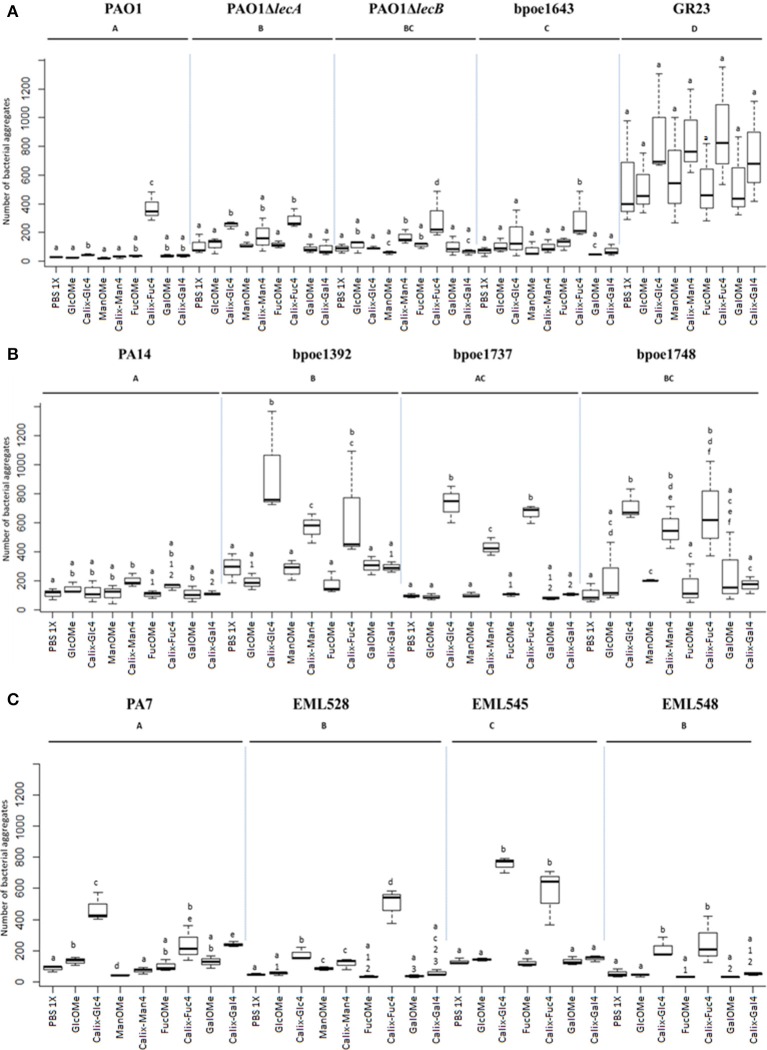
**Box-plots of *P. aeruginosa* cells aggregation values obtained from tetravalent calixarene glycoclusters (Calix-Glc_4_, Calix-Man_4_, Calix-Fuc_4_, and Calix-Gal_4_)**. Number of cell aggregates (>20 μm^2^) were measured by FlowSight cytometry, and compared with those obtained after exposure to methyl glycosides (GlcOMe, ManOMe, FucOMe, and GalOMe). These assays were performed on *P*. *aeruginosa* strains of **(A)** LecB cluster I (PAO1, its isogenic mutants PAO1Δ*lecA* and PAO1Δ*lecB*, bpoe1643 and GR23), **(B)** cluster II (PA14, bpoe1392, bpoe1737 and bpoe1748), and **(C)** cluster III (PA7, EML528, EML545 and EML548) (according to Figure 1). Aggregation levels were recorded from 20,000 cellular events. All tests were at least triplicated. Letter codes indicate the statistical significance of the tests. A same letter code indicates the absence of significant difference between the data sets (*p* > 0.05), and a distinct letter code indicates significant differences (*p* < 0.05). Each black bar in a box shows the median value in terms of number of aggregates obtained for all the tests that were performed.

Global incidence of lectin types on bacterial aggregation was investigated by analyzing the number of aggregates (>20 μm^2^) in a PBS solution of *P. aeruginosa* after exposure to various ligands. Calix[4]arene glucosylated (Calix-Glc_4_), mannosylated (Calix-Man_4_), fucosylated (Calix-Fuc_4_), and galactosylated (Calix-Gal_4_) glycoclusters (Supplementary Figure S1) at 100 μM were added to a panel of PBS-washed *P. aeruginosa* cells representative of the observed LecA/LecB types combinations (Figure 7). The aggregation values were compared with those obtained from cells exposed to PBS or methyl glycosides (GlcOMe, ManOMe, FucOMe, and GalOMe; Supplementary Figure S1).

Considering LecA composition to be quite conserved among *P. aeruginosa*, it is its absence which should lead to differences in the aggregation assays involving galactosylated ligands. To verify this point, a PAO1Δ*lecA* mutant (Boukerb et al., 2014) was included in the assays. This mutant did not produce significant aggregates while being exposed to galactosylated glycoclusters (*p* > 0.05; Figure 7A). However, this calix[4]arene was also poorly efficient at inducing aggregation with wildtype PAO1 cells. Similarly, a PAO1Δ*lecB* mutant was analyzed to investigate changes after exposure to fucosylated and mannosylated glycoclusters. This PAO1Δ*lecB* mutant showed lower aggregation than those obtained with PAO1 exposed to Calix-Fuc_4_ (*p* < 0.05; Figure 7A). These results are in line with an involvement of LecB in the aggregation phenotype. Surprisingly, higher aggregation was observed for this mutant compared to the wild-strain exposed to Calix-Man_4_ (*p* < 0.05; Figure 7A).

Strains harboring various LecB types were tested in these aggregation assays with calix-Man_4_, calix-Fuc_4_, calix-Gal_4_, and calix-Glc_4_. LecB cluster I strains (PAO1 and bpoe1643) showed significantly higher numbers of aggregates when exposed to calix-Fuc_4_ than to monovalent FucOMe (Figure 7 and Supplementary Figure S12). Cluster II strains were much more reactive toward glycoclusters and yielded significantly higher numbers of aggregates with Calix-Man_4_, Calix-Fuc_4_, and Calix-Glc_4_ (Figure 7 and Supplementary Figure S12). However, numbers of aggregates were always much lower for strain PA14 (ST253). This behavior appeared specific of this strain because bpoe1392 which is also part of ST253 showed very strong aggregation responses toward the tested glycoclusters (Figure 7). LecB cluster III strains yielded significantly higher numbers of aggregates when exposed to Calix-Fuc_4_ and Calix-Glc_4_ than monovalent sugars (Figure 7 and Supplementary Figure S12). However, significant differences could not be obtained using Calix-Man_4_ even though, in average, higher numbers were measured. Interestingly, for cluster II and III strains, glucosylated glycoclusters were found inducing aggregation, and sometimes at levels higher than those observed with fucosylated, mannosylated, or galactosylated glycoclusters (Supplementary Figure S12). All these datasets are in line with a role of lectins in *P*. *aeruginosa* aggregation properties. However, other adhesins or cellular products appeared to be involved.

## Discussion

Recent studies demonstrated the great potential of sugar solutions and glycoclusters in the prevention of *P. aeruginosa* infections (e.g., Chemani et al., 2009; Boukerb et al., 2014). However, these investigations have, so far, been done without considering the great diversity observed among this bacterial species, and the impact of naturally selected amino acid changes on the specificity and affinity of these lectins for their ligands. Here, the analysis of a significant collection of clinical and outdoor *P. aeruginosa* isolates revealed genetic and genomic changes at the *lecA* and *lecB* loci that could affect the efficacy of such glyco-therapies.

In this work, loss and IS disruption of *lecA* among *P. aeruginosa* genotypes were observed but a higher genetic drift and likelihood of recombination were computed for *lecB* than *lecA* CDS. PyMol analyses of LecA types suggested low or no incidence of the detected amino acid changes on its functioning. More significant changes were detected among LecB types, and suggested modifications in the interactions with the ligands, and changes in the binding spectra. *P. aeruginosa* LecB types were divided into three clusters (I, II, and III). Cluster I variants (PAO1-related strains) were not likely to have sugar affinities different from those reported so far. Cluster II (PA14-related strains) variants showed a Ser23Ala modification which was previously shown to double affinities toward fucose (Adam et al., 2007). LecB cluster III variants (PA7-related strains) showed modifications likely affecting the LecB sugar binding site and its oligomerization. These latter changes led us to further characterize the structure, specificities, and affinities of a LecB from this cluster i.e., the one of strain PA7.

LecB_PA7_ bound to the same oligosaccharide epitopes as LecB_PAO1_ on the tested glycan array but showed higher affinity for some organizations such as the ones of blood group O/H over those of A and B, and those of Lewis^x^ (Le^x^) and Lewis^a^ (Le^a^) epitopes. Titration microcalorimetry analyses of LecB_PA7_ showed methyl-fucoside and Lewis^a^ to have a stronger affinity (*K*_d_ ~ μM) than methyl-mannoside (*K*_d_ = 73 μM). Nevertheless, since LecB_PA7_ binds as efficiently to mannose as LecB_PAO1_, but less efficiently to fucoside, this confirms its lower specificity for fucosylated saccharides. When analyzing the thermodynamics of binding, the lower affinity of FucOMe was not related to a less efficient binding in the LecB_PA7_ pocket. Indeed, the enthalpy contribution was higher than for LecB_PAO1_ but the entropy barrier was much stronger, pointing out for a different contribution of water molecules. Structural analysis of LecB_PA7_ complexed with Lewis^a^ tetrasaccharide validated this inference. This structure was compared with the one of the LecB_PAO1_/Lewis^a^ complex (Perret et al., 2005) that shares the same overall organization. Fucose residues have the same orientation in both lectins but galactose (Gal) and GlcNAc of Lewis^a^ are slightly reoriented. In LecB_PA7_, Ser97, instead of Gly97 in LecB_PAO1_, can establish a hydrogen bond with one of the two structural water molecules of the binding site. The resulting disturbance of the water molecule network in this area correlates with the stronger entropy barrier measured for LecB_PA7_ when binding to sugars than LecB_PAO1_. Furthermore, the Ala24Gly modification in LecB_PA7_ generated an additional hydrophobic contact close to the CH_2_ group at C6 of the GlcNAc residue. These two major modifications likely contributed to the changes observed over fucose and Lewis^a^ affinities.

Further analyses of the diversity of Lec types in *P. aeruginosa* and comparisons with the phylogenetic allocations of concatenated DNA sequences from the MLST scheme of the *P. aeruginosa* strains used in this study allowed inferring dominant LecA and LecB types in the collection. Nine combinations of LecA/LecB types were recorded in the PAO1 cluster, four in PA14 one, and three in PA7 one. The PA7 and alike *lecB* types were found to have a restricted distribution. Some peculiar combinations suggesting either genetic convergence or genetic recombination in the *P. aeruginosa* global population were identified. In fact, LecA types 2 and 4 were found restricted to the PAO1 phylogenetic group but types 1 and 3 were recruited by both the PAO1 and PA14 groups. Nevertheless, the *lecA* loci did not appear to have undergone significant changes by recombination in the *P. aeruginosa* population. However, the *lecB* loci were found to have been impacted by recombination events. In fact, LecB types 5 and 6 were found detected among highly distant lineages in the PAO1 (cluster I) and PA14 (cluster II) phylogenetic groups.

The full genome of strains EML528, EML545, and EML548 closely related to PA7 were sequenced and annotated (Boukerb et al., 2015) in order to better appreciate the importance of genomic instability at the vicinity of *lecA* (or its matching RGP in PA7 genomes i.e., RGP26) and *lecB* loci. Maps of these genomes were compared, and two major regions of genomic instability were identified (named α and β). Several kb surrounding *lecA* (or its matching RGP26) and *lecB* were subjected to deeper investigations in terms of DNA signatures involved in genomic plasticity. The *lecA* area was found to contain more tRNA gene sequences (tRNA^Leu^, tRNA^Cys^, tRNA^Gly^, and tRNA^Ser^) than the *lecB* one (tRNA^Arg^). These tRNA genes are preferential targets for phages, genomic islands (GI) and integrative and conjugative elements (ICE). However, the tRNA^Arg^ CDS of the *lecB* genomic region did not appear to have been targeted by such elements. In fact, no phage DNA and integrase CDS could be detected in the analyzed *lecB* area even though four RGP could be detected. Still, the *lecB* stop codon was found matching a border of RGP96 which was found containing variable but short inserts (between 600 and 900 bp). These changes in the terminator region could be affecting the *lecB* expression levels. This observation was indicative of genomic instability at the proximity of *lecB* but no strain deleted of this gene could be detected in our collection. One strain, known as KK72 and isolated from a CF patient, was found lacking a 24 kb-long DNA segment containing *lecB* (Region KK_1, PA3357 to PA3391; Lucchetti-Miganeh et al., 2014). This strain showed a poor ability at forming biofilm and had an altered twitching phenotype.

Genomic instability was observed at the vicinity of *lecA*. Its promoter and terminator regions were found to be conserved between *P*. *aeruginosa* strains but to match the borders of RGP26. RGP26 in PA7 is described as encoding a prophage (Roy et al., 2010). Two other RGP, RGP90, and RGP94, were also found colonized by phage DNA, suggesting the *lecA* area to be frequently challenged by phage attacks. In fact, RGP90 of strain EML545 harbored conserved sequences with a transducible temperate bacteriophage (named B3) previously identified in *P*. *aeruginosa* (Braid et al., 2004). The EML545 ϕB3 homolog showed multiple rearrangements in line with the idea that tailed phages are genetic mosaics arising by the exchange of functional modules within a diverse genetic pool (Krylov, 2014). RGP94, which is physically linked to a tRNA^Ser^ CDS, showed variable CDS contents among the analyzed genomes but was colonized by a lytic bacteriophage named ϕP52 in strain EML548. This bacteriophage is part of the *Podoviridae* family found to cause lysis of verona integron-encoded metallo-beta-lactamase-producing, carbapenem-resistant, *P. aeruginosa* (Jeon et al., 2012). However, several CDS involved in DNA replication and modification, and host cell lysis were apparently lacking from this homolog. Another tRNA CDS of the *lecA* area was also targeted by a mobile element i.e., PAGI-2. This is a 105 kb-long genomic island previously described among clone C (Larbig et al., 2002; Klockgether et al., 2004). A PAGI-2 element was also detected among strain EML548 but outside the investigated *lec* regions. These PAGI-2 were full GIs showing all CDS required for their chromosomal integration or horizontal transfer (Larbig et al., 2002).

The above observations clearly demonstrated that genetic and genomic diversifications among *P. aeruginosa* will affect the lectin-ligand affinity profiles of a strain. One could thus question the use of glycomimetics in the prevention of *P. aeruginosa* infections. Still, several other adhesins play part in the oligosaccharide-mediated recognition and adhesion to host cells (e.g., Duque et al., 2013). In fact, *in silico* searches of PAO1, PA7 and the closely related genomes reported here can lead to a long list of CDSs likely involved in the expression/synthesis of adhesins e.g., *znuA, cupA4, cupB6, cupE6, fimA, pilE, fliC, and fliD* (data not shown). In order to clarify this situation, synthetized tetravalent glycoclusters built over a calixarene backbone were used to evaluate their ability at favoring aggregation of *P. aeruginosa* cells. This assay detected a significant effect of Calix-Gal_4_ on strain PA7 (which is not having *lecA*) confirming the likely involvement of other adhesins/processes on the generation of these aggregates. It is noteworthy that, in these assays, Calix-Fuc_4_ was found the most efficient glycocluster for inducing aggregation on a large panel of *P. aeruginosa* strains. Furthermore, LecB cluster II (PA14 and related strains) and III strains (PA7 and related strains) were much more reactive toward the tested glyco-clusters and yielded significantly higher numbers of aggregates with Calix-Fuc_4_ and Calix-Glc_4_ than cluster I strains (PAO1 and related strains). These data sets are in line with a role of lectins in *P. aeruginosa* aggregation properties. High concentration of Calix-Fuc_4_ could thus be efficient at preventing lung colonization not only of strain PAO1, as reported by Boukerb et al. (2014), but of most *P. aeruginosa* strains.

## Conclusion

This work showed genomic and genetic changes at the *lec* loci suggesting ongoing adaptive processes among *P. aeruginosa* which can lead to their loss or changes in their affinities toward sugar ligands. The PA7 clade was found to have counter-selected the *lecA* locus while maintaining the *lecB* one. Significant differences with LecB_PAO1_ were recorded and shown to have affected LecB_PA7_ affinities for sugar ligands. These changes appeared related to the early divergence observed among the *P. aeruginosa* radiation between the PAO1/PA14 and PA7 lineages. Genetic exchanges among the PA7 clade at the *lecA* and *lecB* loci were not detected but analyses of their neighboring sequences showed several RGP with integration of exogenous genetic elements including the well-described PAGI-2 at RGP29 of strain EML528. This is indicative of genetic exchanges among the PA7 clade. All these changes did not appear to have significantly impacted the interactions with Calix-Fuc_4_. This glyco-cluster was found to significantly increase aggregation of *P. aeruginosa* cells from the three main clades (PAO1, PA14, and PA7 clades), confirming its great potential as a glycomimetic. High concentration of this glycomimetic could thus be efficient at preventing lung colonization by most *P. aeruginosa* strains.

## Author contributions

Conceived and designed experiments: AB, SV, AI, and BC. Performed the experiments: AB, AD, SR, RT, AR, LR, SV, AV, and BC. Analyzed the data: AB, AI, SR, LC, SB, ADJ, AV, BC. Wrote the paper: AB, AI, BC. All authors read and approved the final manuscript.

## Funding

This work was partly funded by Anses project “pyo-eau” #2011/1/137 of the “Programme Environnement-Santé-Travail” (French Ministers in charge of ecological and environmental issues). We thank the CNRS, Université Lyon 1, VetAgro Sup, Cluster “Infectiology” of the Rhône-Alpes region, and Labex IMU (Intelligence des Mondes Urbains) (France) for having funded parts of this work. Parts of this work were also funded by the French Fond Unique Interministériel (managed by Oseo and DGCIS), FUI anti-pyo, Labex ARCANE (ANR-11-LABX-0003-01), COST Action CM-1102 MultiGlycoNano, and the Conseil Régional Rhône-Alpes. AB, AI, and BC acknowledge support from GDR *Pseudomonas*.

### Conflict of interest statement

The authors declare that the research was conducted in the absence of any commercial or financial relationships that could be construed as a potential conflict of interest.
