# Supplementary material for: Genomic Rearrangements and Functional Diversification of lecA and lecB Lectin-Coding Regions Impacting the Efficacy of Glycomimetics Directed against Pseudomonas aeruginosa
Source: Front Microbiol. 2016 May 31;7:811. doi: 10.3389/fmicb.2016.00811 (PMC4885879; doi:10.3389/fmicb.2016.00811)
Supplement: Supplementary file 1 [file Table1.PDF]

Genomic rearrangements and functional diversification of *lecA* and *lecB* lectin-coding regions impacting the efficacy of glycomimetics directed against *Pseudomonas aeruginosa*

Amine M. Boukerb, Aude Decor, Sébastien Ribun, Rachel Tabaroni, Audric Rousset, Loris Commin, Samuel Buff, Anne Doléans-Jordheim, Sébastien Vidal, Annabelle Varrot, Anne Imberty and Benoit Cournoyer

Correspondence : B. Cournoyer, UMR CNRS 5557 Microbial Ecology, Main building, aisle 3, 1st floor, 69280 Marcy-L'Etoile, France. Tel. (+33) 478 87 56 47. Fax. (+33) 472 43 12 23. Email: benoit.cournoyer@vetagro-sup.fr

Supplementary Table S1. LecA types recorded among the *P. aeruginosa* collection used in this work.

	***P. aeruginosa* strains**			
**LecA types**	**River/CSO (bpoe)** [a]	**WWTL (poe)** [b]	**CF (GR)** [c]	**Non-CF** [d]
**Type 1 (PAO1, PA14)**	1004, 1009, 1069, 1075, 1113, 1234, 1243, 1293, 1356, 1357, 1359, 1360, 1361, 1362, 1363, 1377, 1392, 1397, 1582, 1586, 1613, 1615, 1633, 1636, 1642, 1645, 1646, 1647, 1648, 1657, 1704, 1711, 1714, 1721, 1728, 1737, 1743, 1768, 1807, 1823, 1830	501, 523, 528, 529, 530, 548, 550, 555, 557, 560, 562, 565, 568, 574	1, 2, 5, 6, 7, 8, 9, 10, 12, 13, 15, 16, 17, 18, 20, 21, 23, 24, 26, 27, 28, 29, 32, 33, 34, 35, 37, 38	209, 247, 299, 311, 367, 373, 396, 431, 450, 520, 530, 559, 594, 629, 727, 773, 795, 810, 818, 850, 865, 980, 1005, 1011, 1039, 1040, 1123, 1135, 1147, 1172, 1245, 1273, 1368, poeE6
**Type 2**	1058, 1079, 1112	546	3	376
**Type 3**	1398, 1400, 1403, 1404, 1406, 1407, 1748	525, 532, 561, 567	14	194, 942, 1020, 1093, 1239
**Type 4**	1643			
**Type 5**	1649			
**IS disrupted (unclassified)**		1196 (IS*Psp4* – position 288* *tpnA* forward) 1293 (IS*Psp4* – position 288* *tpnA* forward)		

[a] Strains isolated from water, sediments, epilithic biofilm and submerged aquatic vegetation from a peri-urban river impacted by a combined sewer overflow (CSO) (from Petit et al., at the eml-brc). [b] 501 to 560: strains isolated from the wastewater treatment lagoon of Montracol (Ain, France). 561 to 568: strains isolated from the wastewater treatment lagoon of Saint-Paul de Varax (Ain, France). 574 : strain isolated from the wastewater treatment lagoon of Buellas (Ain, France) (Petit et al., 2013). 1196 and 1293: strains isolated from the wastewater treatment lagoon of Montracol (Lavenir et al., 2014). [c] Strains isolated from sputa of cystic fibrosis patients (CF) at Michalon Hospital (Grenoble, France) (Doléans-Jordheim et al., 2009).[d] Strains isolated from non-CF patients (sputa, catheter, otitis, peritoneal fluid, operating, pachydermoperiostosis, bronchial aspiration, blood culture, urine, cerebrospinal fluid, and broncho-alveolar liquid) provided by the French "Collège de Bactériologie, de Virologie et d'Hygiène des Hôpitaux" (Paris, France). PAO1: burn wound isolate (Holloway, 1955). PAO1Δ*lecA* and PAO1Δ*lecB* (Boukerb et al., 2014). PA14: plant isolate (Lee et al., 2006). *Positions according to *lecA* numbering of PAO1 (PA2570).
