# Supplementary material for: Genomic Rearrangements and Functional Diversification of lecA and lecB Lectin-Coding Regions Impacting the Efficacy of Glycomimetics Directed against Pseudomonas aeruginosa
Source: Front Microbiol. 2016 May 31;7:811. doi: 10.3389/fmicb.2016.00811 (PMC4885879; doi:10.3389/fmicb.2016.00811)
Supplement: Supplementary file 2 [file Table2.PDF]

Supplementary Table S2. LecB types recorded among the *P. aeruginosa* collection used in this work.

LecB types	***P. aeruginosa*** **strains**			
	**River/CSO (bpoe)** [a]	**WWTL (poe)** [b]	**CF (GR)** [c]	**Non-CF** [d]
**Type 1 (PAO1)**	1004, 1009, 1058, 1069, 1075, 1079, 1112, 1113, 1234, 1243, 1293, 1356, 1357, 1359, 1360, 1361, 1362, 1363, 1377, 1582, 1586, 1613, 1615, 1633, 1636, 1642, 1643, 1645, 1646, 1647, 1648, 1649, 1657, 1711, 1743, 1807, 1830	501, 529, 530, 546, 548, 550, 557, 560, 562, 565, 574, 1196, 1293	2, 3, 5, 6, 7, 8, 9, 10, 12, 13, 15, 16, 17, 18, 20, 21, 24, 26, 27, 28, 29, 32, 33, 34, 35, 37, 38	209, 299, 311, 367, 373, 376, 396, 431, 450, 530, 559, 594, 795, 810, 818, 850, 865, 980, 1005, 1011, 1123, 1147, 1172
**Type 2**			23	
**Type 3**		561	14	
**Type 4**		523, 555		1040
**Type 5**	1704, 1714, 1721, 1728, 1737, 1768, 1823			629, 727, 942
**Type 6**	1398, 1400, 1403, 1404, 1406, 1407, 1748	525, 532, 567, 568	1	194, 520, 1020, 1093, 1239
**Type 7 (PA14)**	1392, 1397	528		247, 773, 1039, 1135, 1245, 1273, 1368, poeE6
**Type 8 (PA7)**				EML528*
**Type 11**				EML545*
**Type 12**				EML548

[a] Strains isolated from water, sediments, epilithic biofilm and submerged aquatic vegetation from a peri-urban river impacted by a CSO (from Petit et al., at the eml-brc). [b] 501 to 560: strains isolated from the wastewater treatment lagoon of Montracol (Ain, France). 561 to 568: strains isolated from the wastewater treatment lagoon of Saint-Paul de Varax (Ain, France). 574 : strain isolated from the wastewater treatment lagoon of Buellas (Ain, France) (Petit et al., 2013). 1196 and 1293: strains isolated from the wastewater treatment lagoon of Montracol (Lavenir et al., 2014). [c] Strains isolated from sputa of cystic fibrosis patients (CF) at Michalon Hospital (Grenoble, France) (Doléans-Jordheim et al., 2009). [d] Strains isolated from non-CF patients (sputa, catheter, otitis, peritoneal fluid, operating, pachydermoperiostosis, bronchial aspiration, blood culture, urine, cerebrospinal fluid, and broncho-alveolar liquid) provided by the French "Collège de Bactériologie, de Virologie et d'Hygiène des Hôpitaux" (Paris, France). PA7: Non-respiratory tract clinical isolate (Roy et al., 2010). * EML: Environmental Microbiology Lyon – Biological Resource Center (http://eml-brc.org).
