# Supplementary material for: Genomic Rearrangements and Functional Diversification of lecA and lecB Lectin-Coding Regions Impacting the Efficacy of Glycomimetics Directed against Pseudomonas aeruginosa
Source: Front Microbiol. 2016 May 31;7:811. doi: 10.3389/fmicb.2016.00811 (PMC4885879; doi:10.3389/fmicb.2016.00811)
Supplement: Supplementary file 3 [file Table3.PDF]

Supplementary Table S3. PCR primers and conditions used for screening or cloning *P. aeruginosa* targeted genes.

Gene	Primer code	Primer sequence (5'→ 3')	Expected size (pb)	Primer annealing (°C)	Reference
ecfX	*ecfX*-F	ATGGATGAGCGCTTCCGTG	528	58	Lavenir et al., 2007
	ecfX-R	TCATCCTTCGCCTCCCTG			Lavenir et al., 2007
lecA	*lecA*-F	CGGAGATCACATATGGCTTGGAAAGG	394	50	Chemani et al., 2009
	lecA-R	CCGAGACAAGTTATCAGG			This study
lecB	*lecB*-F	GGAGATACCATGGCAACACAAGGAG	358	52	Mitchell et al., 2005
	lecB-R	CTAGCCGAGCGGCCAG			This study
	lecB8-F *Nde*I	GGAGATACCATATGGCAACACAAGGAG	358	66	Mitchell et al., 2005
	lecB8-R *Hind*III	TTCCAAGCTTCTAGCCGAGCGG			Mitchell et al., 2005

Thick line = Start and stop codons ; faint line = Restriction sites.
