# Supplementary material for: Genomic Rearrangements and Functional Diversification of lecA and lecB Lectin-Coding Regions Impacting the Efficacy of Glycomimetics Directed against Pseudomonas aeruginosa
Source: Front Microbiol. 2016 May 31;7:811. doi: 10.3389/fmicb.2016.00811 (PMC4885879; doi:10.3389/fmicb.2016.00811)
Supplement: Supplementary file 4 [file Table4.PDF]

Supplementary Table S4. Data collection and refinement statistics for $LecB_{PA7}$ complexed with Lewis[a].

Parameters	***Data or properties***			
Beamline (wavelength, Å)	BM30A/0.9205			
Spacegroup	$P2_1$			
Unit cell dimensions, a, b,c Å	52.79 70.37 54.66 90.00 90.50 90.00			
Resolution (outer shell), Å	42.23-1.75 (1.78-1.75)			
Measured/ Unique reflections	135976/40169			
Average multiplicity	3.4 (2.7)			
R_{merge}	0.061 (0.353)			
R_{pim}	0.058 (0.328)			
Completeness (%)	99.4 (93.5)			
Mean I / σI	13.7 (3.0)			
CC1/2	0.996 (0.796)			
Refinement				
R_{cryst}/ R_{free}	14.75/18.34			
nb reflections/free reflections	38130/1992			
R_{msd} bonds, Å	0.015			
R_{msd} angles,°	1.62			
Rmsd chiral, $Å^3$	0.098			
Atoms (chain)	A	B	C	D
Protein	841	832	829	833
Bfac $Å^2$	9.9	10.2	12.8	13.1
Water molecules	213	179	139	113
Bfac, $Å^2$	21.0	22.2	22.6	23.6
Ligand	52	47	47	36
Bfac, $Å^2$	19.5	18.6	16.7	16.0
Calcium	2	2	2	2
Bfac, Å2	9.7	10.5	15.2	19.6
Ramachandran (Molprobity) Allowed	100 %			
Favored	97.4 %			
Outliers :	0 %			
PDBcode	4UT5			
