# Supplementary material for: Genomic Rearrangements and Functional Diversification of lecA and lecB Lectin-Coding Regions Impacting the Efficacy of Glycomimetics Directed against Pseudomonas aeruginosa
Source: Front Microbiol. 2016 May 31;7:811. doi: 10.3389/fmicb.2016.00811 (PMC4885879; doi:10.3389/fmicb.2016.00811)
Supplement: Supplementary file 5 [file Table5.PDF]

Supplementary Table S5. Comparison of affinities and thermodynamic contributions for the binding of $LecB_{PA7}$ and $LecB_{PAO1}$ to different glycans. Standard deviations lower than 20% were obtained for the ITC experiments.

Ligand	LecB origin	K_D (µM)	ΔG (kJ/mol)	ΔH (kJ/mol)	-TΔS (kJ/mol)
**αMeFuc**	PA7/PAO1	2.2/0.43[a]	-32.2/-36.4	-48.4/-41.3	16.2/4.9
**αMeMan**	PA7/PAO1	73/71[a]	-23.6/-23.7	-16.7/-17.8	-6.8/-5.9
**Lewis A**	PA7/PAO1	2.01/0.29[b]	-32.5/-38.1	-44.4/-35.0	11.9/-3.1

[a] from Sabin et al., (2006).
[b] from Perret et al., (2005).
