# Supplementary material for: Genomic Rearrangements and Functional Diversification of lecA and lecB Lectin-Coding Regions Impacting the Efficacy of Glycomimetics Directed against Pseudomonas aeruginosa
Source: Front Microbiol. 2016 May 31;7:811. doi: 10.3389/fmicb.2016.00811 (PMC4885879; doi:10.3389/fmicb.2016.00811)
Supplement: Supplementary file 6 [file Table6.PDF]

Supplementary Table S6. List of the 40 best hits obtained with Alexa-labelled $LecB_{PA7}$ on mammalian glycan array from the Consortium for Functional Glycomics. Full list available on the CFG web site.

Chart Number	LecB PA7_200ug/ml_16973_Alexa488_v5.2_07/22/14_MA	AvgMean	StDev
543	Fuca1-2Galb1-4GlcNAcb1-3Galb1-4GlcNAcb1-2Mana1-6(Fuca1-2Galb1-4GlcNAcb1-3Galb1-4GlcNAcb1-2Mana1-3)Manb1-4GlcNAcb1-4GlcNAcb-Sp24	19314	1365
471	Fuca1-2Galb1-4(Fuca1-3)GlcNAcb1-2Mana1-6(Fuca1-2Galb1-4(Fuca1-3)GlcNAcb1-2Mana1-3)Manb1-4GlcNAcb1-4(Fuca1-6)GlcNAcb-Sp24	10400	449
69	Fuca1-2Galb1-4(Fuca1-3)GlcNAcb1-3Galb1-4(Fuca1-3)GlcNAcb-Sp0	9931	478
70	Fuca1-2Galb1-4(Fuca1-3)GlcNAcb1-3Galb1-4(Fuca1-3)GlcNAcb1-3Galb1-4(Fuca1-3)GlcNAcb-Sp0	8392	103
74	Fuca1-2Galb1-4GlcNAcb1-3Galb1-4GlcNAcb1-3Galb1-4GlcNAcb-Sp0	7703	251
154	Galb1-4(Fuca1-3)GlcNAcb1-3Galb1-4(Fuca1-3)GlcNAcb1-3Galb1-4(Fuca1-3)GlcNAcb-Sp0	7393	391
73	Fuca1-2Galb1-4GlcNAcb1-3Galb1-4GlcNAcb-Sp0	7334	748
126	Galb1-3(Fuca1-4)GlcNAcb1-3Galb1-4(Fuca1-3)GlcNAcb-Sp0	7219	908
277	Galb1-3(Fuca1-4)GlcNAcb1-3Galb1-3(Fuca1-4)GlcNAcb-Sp0	6982	345
239	Neu5Aca2-3Galb1-3(Fuca1-4)GlcNAcb-Sp8	6981	497
316	Mana1-2Mana1-6(Mana1-2Mana1-3)Mana1-6(Mana1-2Mana1-2Mana1-3)Mana-Sp9	6780	230
330	Neu5Aca2-3Galb1-3(Fuca1-4)GlcNAcb1-3Galb1-3(Fuca1-4)GlcNAcb-Sp0	6532	769
76	Fuca1-2Galb1-4GlcNAcb-Sp8	6086	954
240	Neu5Aca2-3Galb1-3(Fuca1-4)GlcNAcb1-3Galb1-4(Fuca1-3)GlcNAcb-Sp0	6072	472
59	Fuca1-2Galb1-3GalNAcb1-3Gala1-4Galb1-4Glcb-Sp9	5981	354
51	Mana1-6(Mana1-3)Manb1-4GlcNAcb1-4GlcNAcb-Sp13	5968	390
71	Fuca1-2Galb1-4(Fuca1-3)GlcNAcb-Sp0	5945	698
28	(3S)Galb1-3(Fuca1-4)GlcNAcb-Sp8	5943	811
79	Fuca1-3GlcNAcb-Sp8	5930	263
129	Galb1-3(Fuca1-4)GlcNAc-Sp8	5720	195
130	Fuca1-4(Galb1-3)GlcNAcb-Sp8	5682	488
491	(3S)Galb1-3(Fuca1-4)GlcNAcb-Sp0	5631	62
253	Neu5Aca2-3Galb1-4(Fuca1-3)GlcNAcb1-3Galb1-4(Fuca1-3)GlcNAcb1-3Galb1-4(Fuca1-3)GlcNAcb-Sp0	5606	424
128	Galb1-3(Fuca1-4)GlcNAc-Sp0	5360	783
80	Fuca1-4GlcNAcb-Sp8	5192	570
215	Mana1-6(Mana1-3)Mana1-6(Mana1-2Mana1-3)Manb1-4GlcNAcb1-4GlcNAcb-Sp12	5135	195
214	Mana1-2Mana1-2Mana1-6(Mana1-3)Mana-Sp9	5128	408
483	Mana1-6(Mana1-3)Manb1-4GlcNAcb1-4(Fuca1-6)GlcNAcb-Sp19	5029	254
213	Mana1-6(Mana1-3)Mana-Sp9	5021	539
211	Mana1-2Mana1-6(Mana1-3)Mana1-6(Mana1-2Mana1-2Mana1-3)Manb1-4GlcNAcb1-4GlcNAcb-Sp12	4987	685
270	Neu5Aca2-6Galb1-4GlcNAcb1-3Galb1-4(Fuca1-3)GlcNAcb1-3Galb1-4(Fuca1-3)GlcNAcb-Sp0	4942	308
153	Galb1-4(Fuca1-3)GlcNAcb1-3Galb1-4(Fuca1-3)GlcNAcb-Sp0	4938	151
315	Mana1-2Mana1-6(Mana1-3)Mana1-6(Mana1-2Mana1-2Mana1-3)Mana-Sp9	4804	503
65	Fuca1-2Galb1-3GlcNAcb1-3Galb1-4Glcb-Sp8	4760	397
161	Galb1-4GlcNAcb1-3Galb1-4(Fuca1-3)GlcNAcb1-3Galb1-4(Fuca1-3)GlcNAcb-Sp0	4682	577
280	Neu5Gca2-3Galb1-3(Fuca1-4)GlcNAcb-Sp0	4655	80
210	Mana1-6(Mana1-2Mana1-3)Mana1-6(Mana1-2Mana1-3)Manb1-4GlcNAcb1-4GlcNAcb-Sp12	4635	181
67	Fuca1-2Galb1-3GlcNAcb-Sp0	4549	204
72	Fuca1-2Galb1-4(Fuca1-3)GlcNAcb-Sp8	4444	366
