# Supplementary material for: Genomic Rearrangements and Functional Diversification of lecA and lecB Lectin-Coding Regions Impacting the Efficacy of Glycomimetics Directed against Pseudomonas aeruginosa
Source: Front Microbiol. 2016 May 31;7:811. doi: 10.3389/fmicb.2016.00811 (PMC4885879; doi:10.3389/fmicb.2016.00811)
Supplement: Supplementary file 7 [file Table7.PDF]

Supplementary Table S7. Regions of genomic plasticity surrounding the *lecA* locus and identified by ACT comparisons in *P. aeruginosa* PAO1, PA7, and EML strains sequenced in this work.

Location in PAO1 (length in bp)	RGP	Location in PA7 (length in bp / ID% according to PAO1)	Location in EML528 (length in bp / ID% according to PA7)	Location in EML545 (length in bp / ID% according to PA7)	Location in EML548 (length in bp / ID% according to PA7)	Inserted near tRNA	Conserved CDS codes	CDS number and/or inferred function (COG)
2862110..2863981<br>(1871)	**RGP90**	2717953..2717952<br>(0 / 0%)	2740069..2740068<br>(0 / 0%)	2539106..2537236<br>(35102 / 0%)	2729558..2727577<br>(1982 / 0%)	no	**PAO1 (PA)**<br>2534-2535<br>**PA7**<br>No indel found<br>**EML528**<br>No indel found<br>**EML545**<br>140229 - 140189<br>140188 - 140187<br>**EML548**<br>480006-480005	**PAO1, EML548**<br>Transcriptional regulator (0583)<br>Oxidoreductase (0667)<br><br>**EML545**<br>In order: ORF 3, 5, 7-10, 16, 23-41, and 44-58 of Bacteriophage B3 (Supplementary Figure S10)
2877475..2878918<br>(1443)	**RGP91**	2704472..2703030<br>(1443 / 98%)	2725480..2724038<br>(1443 / 99%)	2523814..2522642<br>(1173 / 0%)	2714173..2713001<br>(1173 / 0%)	no	**PAO1 (PA)**<br>PA2546-2547<br>**PA7 (PSPA7)***<br>2691-2690<br>**EML528***<br>140493-140492<br>**EML545**<br>140176<br>**EML548**<br>470136	**PAO1, PA7, EML528**<br>Ring-cleaving dioxygenase (0346)<br>Transcriptional regulator (0583)<br><br>**EML545**<br>HP (3422)<br>**EML548**<br>HP (3422)
2904733..2905942<br>(1209)	**RGP26[£]**	2677165..2668018<br>(9148 / 0%)	2698191..2689043<br>(9148 / 99%)	2496777..2485105<br>(11673 / 98%)	2687137..2675473<br>(11665 / 98%)	tRNA^{Leu}<br>(left)	**PAO1 (PA)**<br>2570-2571<br>**PA7 (PSPA7)**<br>2661-2648<br>**EML528****<br>1404663-140453<br>**EML545****<br>140148-140129<br>+ 2.5kb inserted between PASPA7_2657 and 2656<br>(140144-140138)<br>**EML548****<br>470107-470088<br>+ 2.5kb inserted between PASPA7_2657 and 2656<br>(470103-470097)	**PAO1**<br>PA lectin A<br>**PA7, EML528**<br>2661-2650: HP<br>2649/2648: Phage integrase family proteins (no/0582)<br><br>**EML545**<br>insertion:<br>140144, 140141-140138: HP<br>140143/140142: Lytic enzymes (3772/3179)<br><br>**EML548**<br>insertion:<br>470103, 470101-470097: HP<br>470102 / 470101: Lytic enzymes (3772/3179)
2916749..2917152<br>(403)	**RGP92**	2657270..2656322<br>(949 / 0%)	2678298..2677348<br>(949)	2474353..2471430<br>(2923 / 0%)	2664727..2661827<br>(2901 / 0%)	no	**PAO1**<br>No indel found (+ 403 nt)<br>**PA7 (PSPA7)**<br>2635<br>**EML528***<br>140437<br>**EML545**<br>140116-140115<br>**EML548*****<br>470077-470076	**PA7, EML528**<br>HP<br><br>**EML545, EML548**<br>Fimbrial protein (3539)<br>Transcriptional regulator, MerR family (0789)
2918196..2918556<br>(360)	**RGP72[¥]**	2655278..2654441<br>(838 / 0%)	2676307..2675469<br>(838 / 99%)	2470411..2469576<br>(836 / 98%)	2660788..2659954<br>(835 / 98%)	tRNA^{Cys}<br>(left)	**PAO1**<br>2581<br>**PA7 (PSPA7)**<br>2633-2632<br>**EML528****<br>140435-140434<br>**EML545****<br>140113-140112<br>**EML548****<br>470074-470073	**PA7, EML528, EML545, EML548**<br>EF hand domain-containing protein<br>HP

Continued next page

Table S7. cont'd

Location in PAO1 (length in bp)	RGP	Location in PA7 (length in bp / ID% according to PAO1)	Location in EML528 (length in bp / ID% according to PA7)	Location in EML545 (length in bp / ID% according to PA7)	Location in EML548 (length in bp / ID% according to PA7)	Inserted near tRNA	Conserved CDS codes	Gene class /COG
2918812..2919524<br>(712)	**RGP72**[¥]	2654166..2641296<br>(12870 / 0%)	2675194..2662324<br>(12870 / 99%)	2469302..2451833<br>(17470 / 99%)	2659679..2642206<br>(17474 / 99%)	tRNA^{Cys}<br>(right)	**PAO1**<br>2582<br>**PA7 (PSPA7)**<br>2630-2621<br>**EML528****<br>140435-140426<br>**EML545****<br>140113-140099<br>+ 4.8kb inserted between PASPA7_2622 and 2621<br>(140104-140100)<br>**EML548****<br>470074-470058<br>+ 4.8kb inserted between PASPA7_2622 and 2621<br>(470064-470060)	**PA7, EML528**<br>TetR family transcriptional regulator (1309)<br>Beta-glucosidase (2723)<br>Glycine betaine transmethylase<br>HP<br>Sulfatase (3119)<br>HP<br>Arylsulfatase (3119)<br>Sulfatase-modifying factor (1262)<br>Sugar transporter, glycoside-pentoside-hexuronide (2211)<br>**EML545, EML548**<br>Insertion:<br>140104/470064: 2-keto-3 dexygalactono-kinse (3734)<br>140102/470062: 2-oxo-3-deoxygalactoct-onate-6-phosphate aldolase (0800)<br>140101/470061: galactonate dehydratase (4948)<br>140100/470060: Short-chain dehydrogenase/ reductase SDR (1028)
2919573..2923218<br>(3645)	**RGP27**[£]	2640759..2635979<br>(4781 / 0%)	2657003..2551974<br>(105029 / 99%)	2451291..2446420<br>(4872 / 99%)	2641669..2636809<br>(4861 / 99%)	tRNA^{Gly}<br>(left)	**PAO1 (PA)**<br>2583-2584<br>**PA7 (PSPA7)**<br>2620-2616<br>**EML528 (PAGI-2)**<br>140420-140306<br>**EML545****<br>140098-140095<br>**EML548****<br>470057-470054	**PAO1**<br>PA2583: Sensor/response regulator hybrid (0642)<br>**PA7, EML545, EML548**<br>PSPA7_2620: Thioredoxin domain-containing protein (3531)<br>PSPA7_2619: HP (4319)<br>PSPA7_2618: Putative HTH-type transcriptional regulator YafC (0583)<br>PSPA7_2617: EAL domain-containing protein (4943)<br>**EML528 (RGP29)**<br>(See Supplementary Figure S9A)
2929523..2933615<br>(4092)	**RGP93**	2629633..2629525<br>(109 / 0%)	2545706..2545598<br>(108 / 100%)	2440074..2439966<br>(109 / 99%)	2630463..2630356<br>(108 / 98%)	no	**PAO1 (PA)**<br>2589-2590<br>**PA7**<br>No indel found (+ 108 nt)<br>**EML528**<br>No indel found (+ 108 nt)<br>**EML545**<br>No indel found (+ 109 nt)<br>**EML548**<br>No indel found (+ 108 nt)	**PAO1**<br>HP (0477)<br>HP (1629)
2936423..2947544<br>(11121)	**RGP94**	2626730..2626598<br>(132 / 0%)	2542802..2542670<br>(133 / 98%)	2437171..2437039<br>(133 / 98%)	2627560..2578071<br>(49490 / 98%)	tRNA^{Ser}<br>(left)	**PAO1 (PA)**<br>PA2594-2603<br>**PA7**<br>No indel found (+ 132 nt)<br>**EML528**<br>No indel found (+ 133 nt)<br>**EML545**<br>No indel found (+ 133 nt)<br>**EML548**<br>470043-460051	**PAO1**<br>PA2594-2696, 2699: HP (0715)<br>PA2597 HP (1960)<br>PA2598, 2600: HP (2141)<br>PA2601 Transcriptional regulator (0583)<br>PA2602 HP (5553)<br>PA2603 Thiosulfate sulfurtransferase (0607)<br><br>**EML548**<br>*Pseudomonas* phage P52: 53% of query cover (see Supplementary Figure S11)

HP = hypothetical protein.
ID = identity between two compared RGP sequences.
nt=nucleotides.
***** Conserved in PAO1.
****** Conserved in PA7.
******* Conserved in EML545.
£ According to Mathee et al., (2008).
¥ According to Roy et al., (2010).
