# Supplementary material for: Genomic Rearrangements and Functional Diversification of lecA and lecB Lectin-Coding Regions Impacting the Efficacy of Glycomimetics Directed against Pseudomonas aeruginosa
Source: Front Microbiol. 2016 May 31;7:811. doi: 10.3389/fmicb.2016.00811 (PMC4885879; doi:10.3389/fmicb.2016.00811)
Supplement: Supplementary file 8 [file Table8.PDF]

Supplementary Table S8. Regions of genomic plasticity (RGP) surrounding *lecB* that were identified by ACT comparisons of *P. aeruginosa* PAO1, PA7 and EML genomes

Location in PAO1 (length in bp)	RGP	Location in PA7 (length in bp / ID% according to PAO1)	Location in EML528 (length in bp / ID% according to PA7)	Location in EML545 (length in bp / ID% according to PA7)	Location in EML548 (length in bp / ID% according to PA7)	Inserted near tRNA	Conserved CDS codes	CDS number and/or inferred function (COG)
3730035..3746077 (16043)	**RGP95**	1829501..1829232 (270 / 0%)	1791408..1791139 (270 / 99%)	1631989..1631720 (270 / 99%)	1706662..1706393 (270 / 98%)	no	**PAO1** 3327-3336 **PA7** No indel found **EML528** No indel found **EML545** No indel found **EML548** No indel found	**PAO1** Non-ribosomal peptide synthetase (NRPS) (1020) FAD-dependent monooxygenase (0654) HP (1020) Short-chain dehydrogenase (1028) Cytochrome P450 (2124) HP (3631) 3-oxoacyl-ACP synthase (0332) Acyl carrier protein (0236) HP (0500) Major facilitator superfamily (MFS) transporter (2814)
3773376..3773986 (611)	**RGP96**	1801932..1800962 (971 / 0%)	1763851..1762624 (1228 / 99%)	1656174..1655204 (971 / 99%)	1679096..1677860 (1237 / 99%)	no	**PAO1** 3362 **PA7** 1768 **EML528**** 110010 **EML545**** 100013 **EML548**** 320030	**PAO1** HP (0477) **PA7, EML528, EML545, EML548** Putative lipoprotein
3778606..3778654 (49)	**RGP58£**	1796344..1796297 (48 / 89%)	1758013..1753597 (4417 / 0%)	1650602..1642408 (8195 / 0%)	1676550..1676193 (49 / 100%)	$tRNA^{Arg}$ (left)	**PAO1** 3366-3368 **PA7** 1761-1764 **EML528** 110006-100293 + 4.4 kb (110004-1100332) **EML545** 100009-90486 + 8.1 kb (100007-90488) **EML548**** 320027-320024	**EML528, EML545** HP
3792164..3793109 (946)	**RGP97**	1782774..1782712 (63 / 0%)	1740075..1740013 (63 / 100%)	1628884..1628822 (63 / 100%)	1659669..1659607 (63 / 100%)	no	**PAO1** 3387 **PA7** No indel found **EML528**** No indel found **EML545**** No indel found **EML548**** No indel found	**PAO1** Beta-ketoacyl reductase (rhlG) (1028)

HP = hypothetical protein.
ID = identity between RGP sequences.
****** Conserved in PA7.
£ According to Mathee et al. (2008).
