# Supplementary material for: Genomic Rearrangements and Functional Diversification of lecA and lecB Lectin-Coding Regions Impacting the Efficacy of Glycomimetics Directed against Pseudomonas aeruginosa
Source: Front Microbiol. 2016 May 31;7:811. doi: 10.3389/fmicb.2016.00811 (PMC4885879; doi:10.3389/fmicb.2016.00811)
Supplement: Supplementary file 9 [file Image1.PDF]

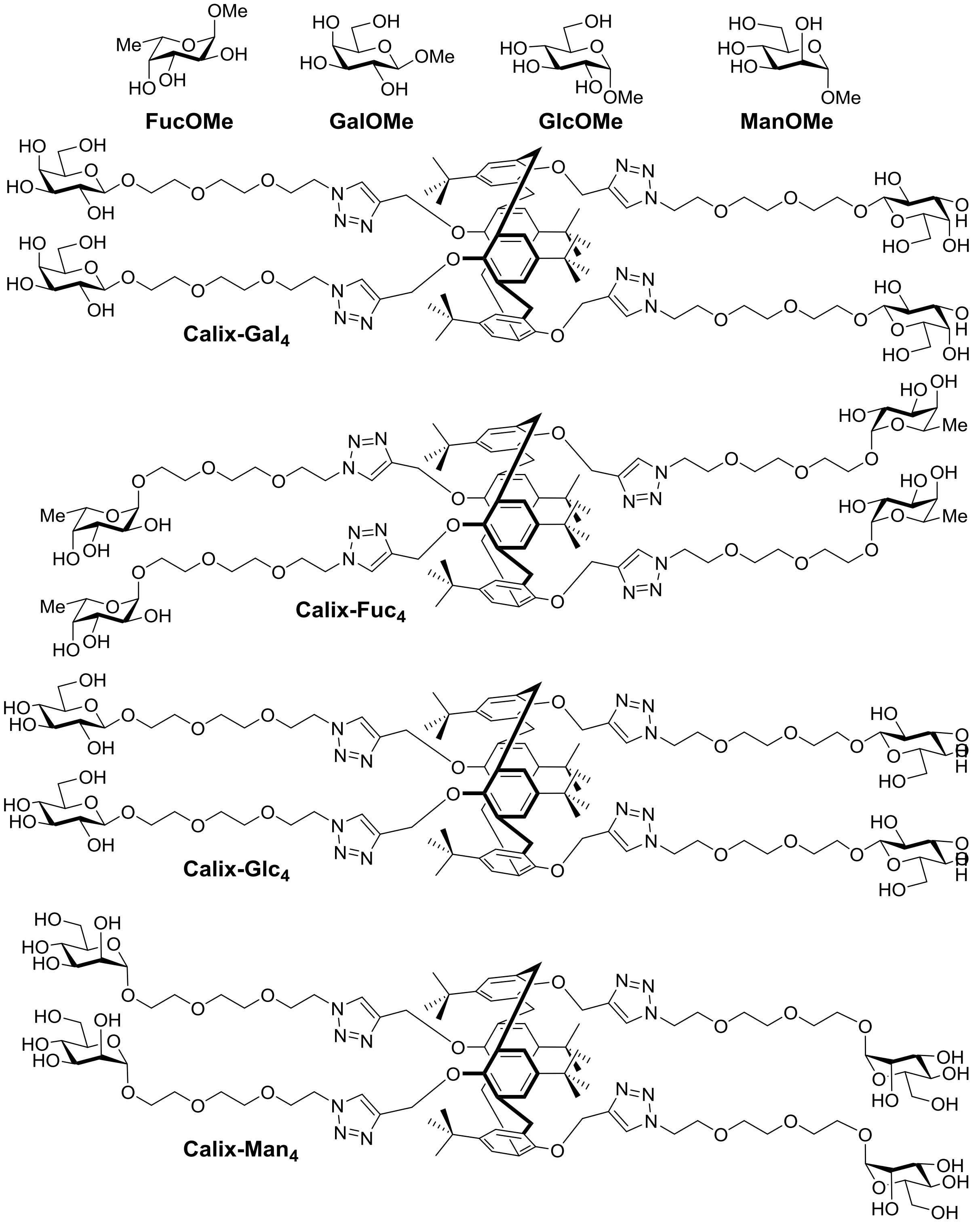

Supplementary Figure S1. Structure of monovalent and tetravalent fucosylated and mannosylated ligands of LecB, and of galactosylated ligands targeting LecA along with glucosylated ligands.
