# Supplementary material for: Genomic Rearrangements and Functional Diversification of lecA and lecB Lectin-Coding Regions Impacting the Efficacy of Glycomimetics Directed against Pseudomonas aeruginosa
Source: Front Microbiol. 2016 May 31;7:811. doi: 10.3389/fmicb.2016.00811 (PMC4885879; doi:10.3389/fmicb.2016.00811)
Supplement: Supplementary file 10 [file Image2.PDF]

(A)

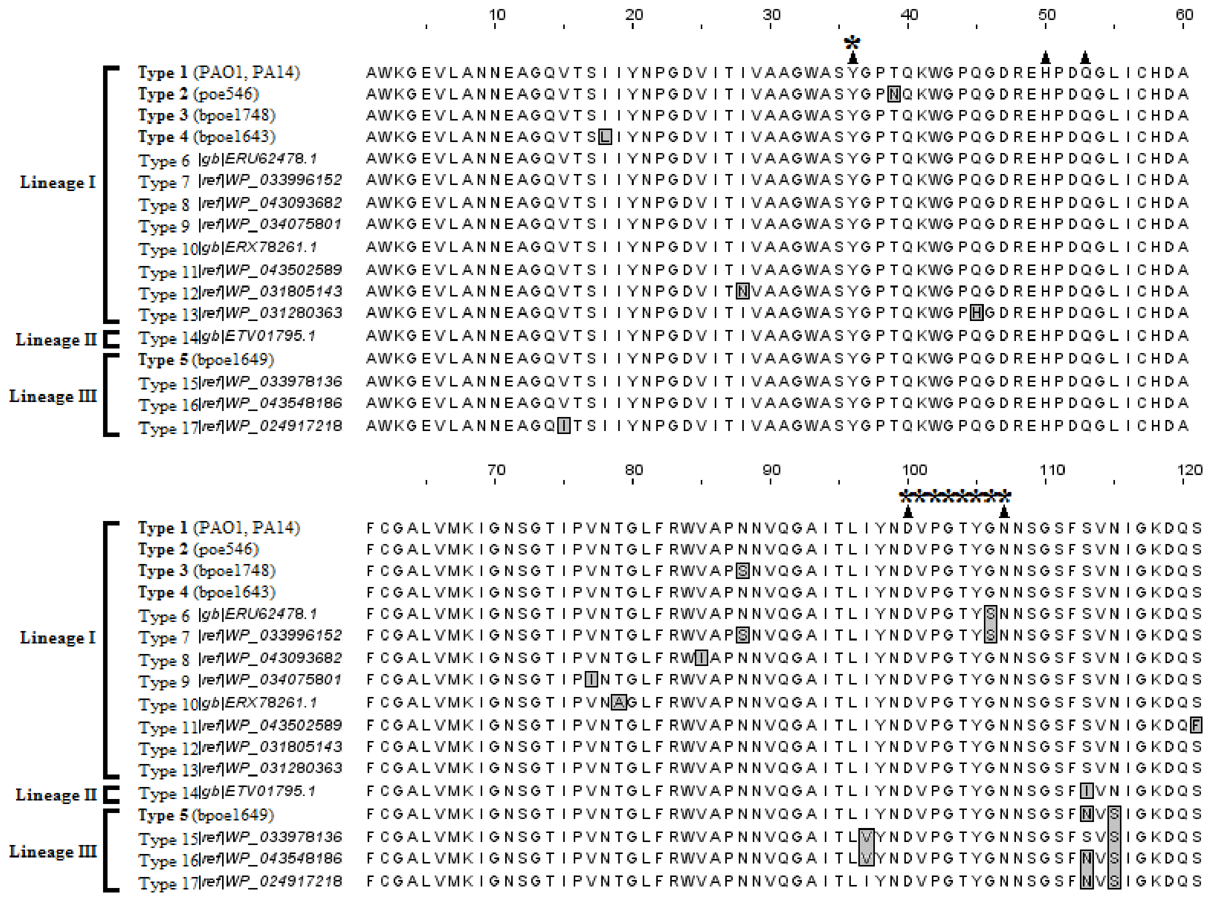

(B)

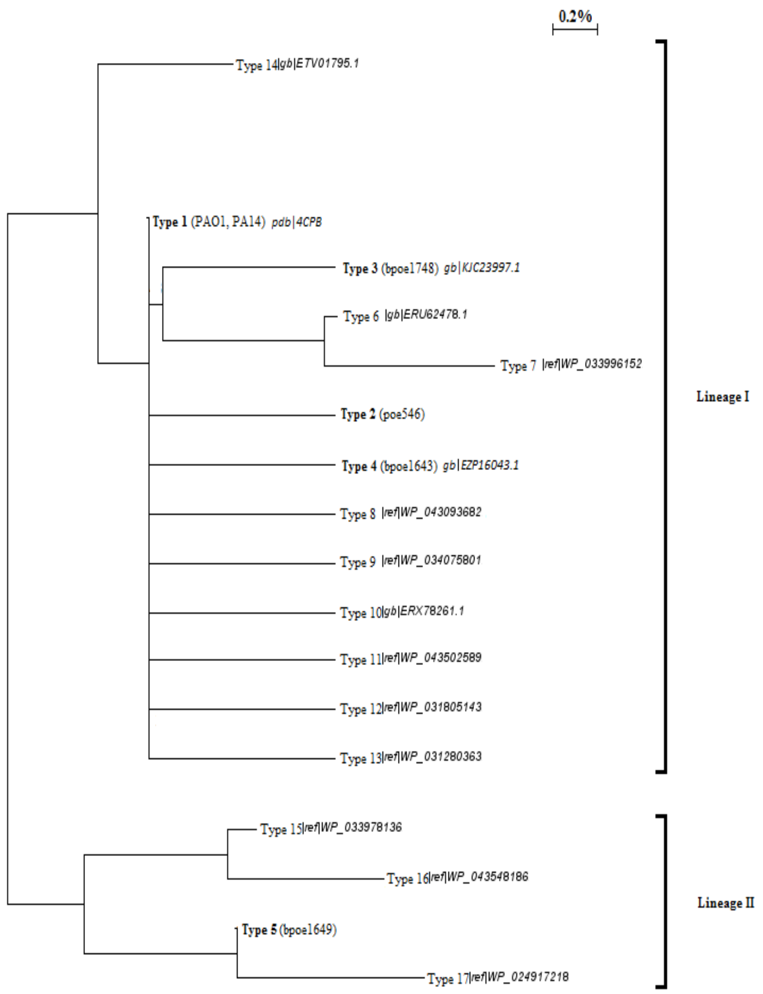

Supplementary Figure S2. LecA types among *P. aeruginosa*. **(A)** Alignment of the seventeen types of LecA amino acid sequences detected in this work (bold text) or recovered from GenBank (faint text). Amino acids different from those of the PAO1 sequence (total of fourteen positions) are boxed and in grey. Triangles indicate the amino acids participating in sugar binding, and asterisks, the amino acids participating in calcium coordination. **(B)** Neighbor-Joining phylogenetic tree of the seventy LecA types. A total of 121 amino acids were analyzed. Horizontal lines represent the divergence % between pairs of sequences. Bootstrap values are indicated on branches.
