# Supplementary material for: Genomic Rearrangements and Functional Diversification of lecA and lecB Lectin-Coding Regions Impacting the Efficacy of Glycomimetics Directed against Pseudomonas aeruginosa
Source: Front Microbiol. 2016 May 31;7:811. doi: 10.3389/fmicb.2016.00811 (PMC4885879; doi:10.3389/fmicb.2016.00811)
Supplement: Supplementary file 11 [file Image3.PDF]

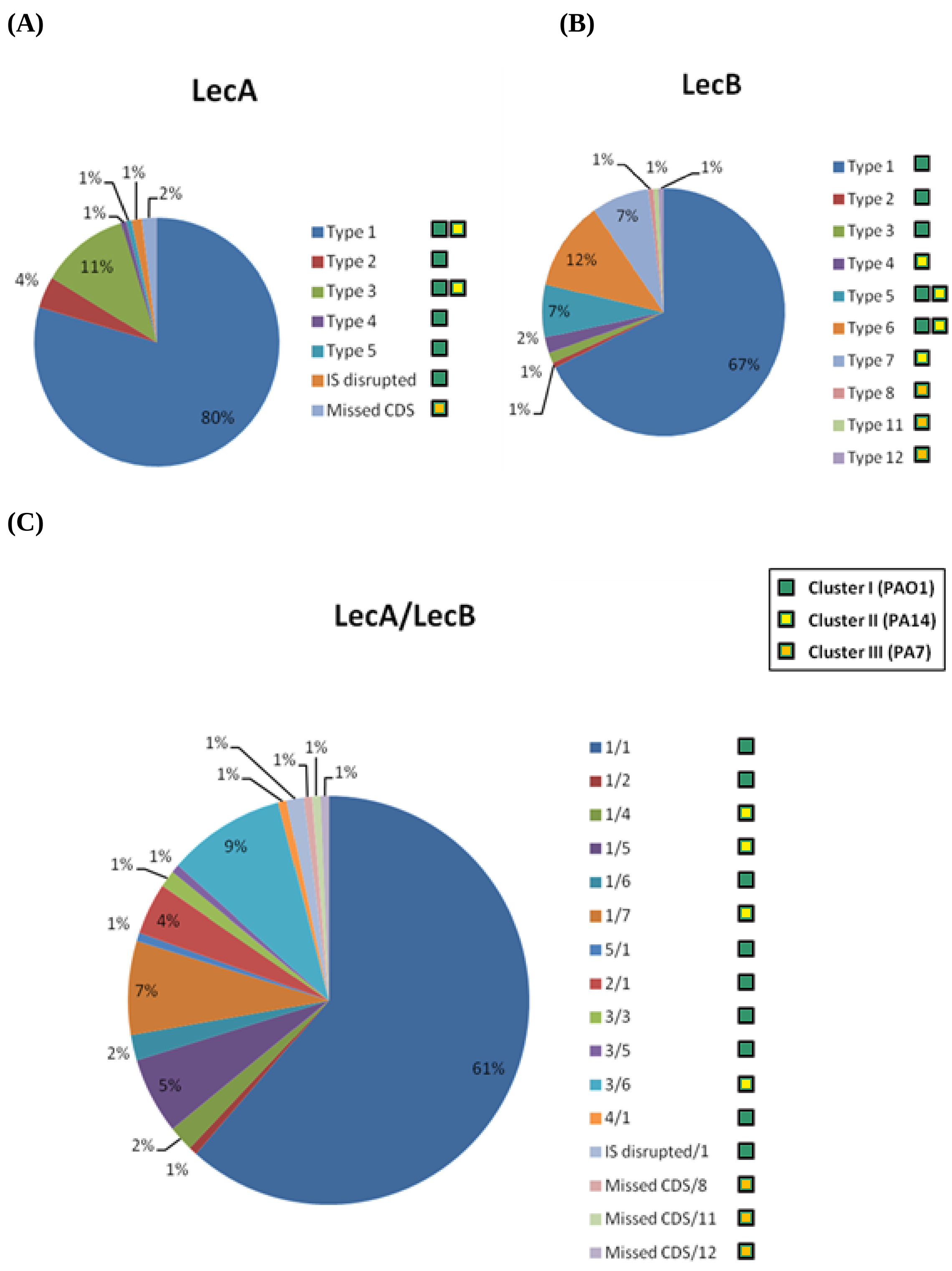

Supplementary Figure S3. Prevalence of LecA and LecB types among *P. aeruginosa* strains analyzed in this work. **(A)** Proportion of LecA types, **(B)** of LecB types, **(C)** and of the LecA and LecB types combinations. Color-codes represent the distribution of LecA, LecB and the LecA/LecB types combinations according to the clusters inferred from the phylogenetic analysis of the MLST dataset (Supplementary Figure S4).
