# Supplementary material for: Genomic Rearrangements and Functional Diversification of lecA and lecB Lectin-Coding Regions Impacting the Efficacy of Glycomimetics Directed against Pseudomonas aeruginosa
Source: Front Microbiol. 2016 May 31;7:811. doi: 10.3389/fmicb.2016.00811 (PMC4885879; doi:10.3389/fmicb.2016.00811)
Supplement: Supplementary file 12 [file Image4.PDF]

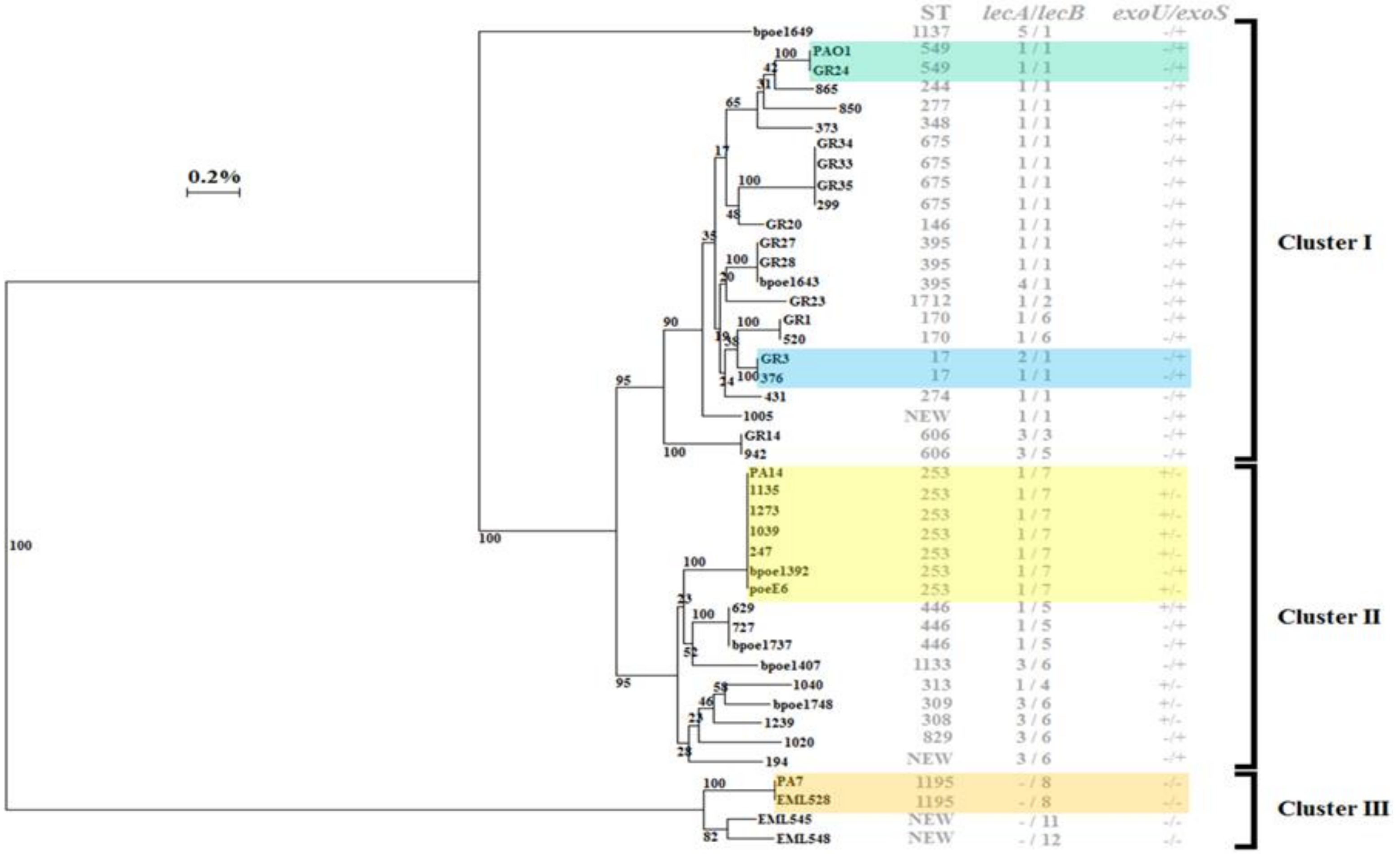

Supplementary Figure S4. Neighbor-joining phylogenetic tree of concatenated *acsA*, *aroE*, *guaA*, *mutL*, *nuoD*, *ppsA* and *trpE* partial gene sequences from representative strains of the *P. aeruginosa* collection. A total of 2882 sites were analyzed using Kimura's 2-parameters model. Horizontal lines represent the divergence % between pairs of sequences. Bootstrap values are indicated. Corresponding sequence types, LecA or LecB identified types and the *exoU*/*exoS* profiles are indicated. LecB clusters are indicated. *lec* types of PAO1 (ST549), Clone C (ST17), PA14 (ST253) and PA7 (ST1195) groups are indicated with different color backgrounds.
