# Supplementary material for: Genomic Rearrangements and Functional Diversification of lecA and lecB Lectin-Coding Regions Impacting the Efficacy of Glycomimetics Directed against Pseudomonas aeruginosa
Source: Front Microbiol. 2016 May 31;7:811. doi: 10.3389/fmicb.2016.00811 (PMC4885879; doi:10.3389/fmicb.2016.00811)
Supplement: Supplementary file 13 [file Image5.PDF]

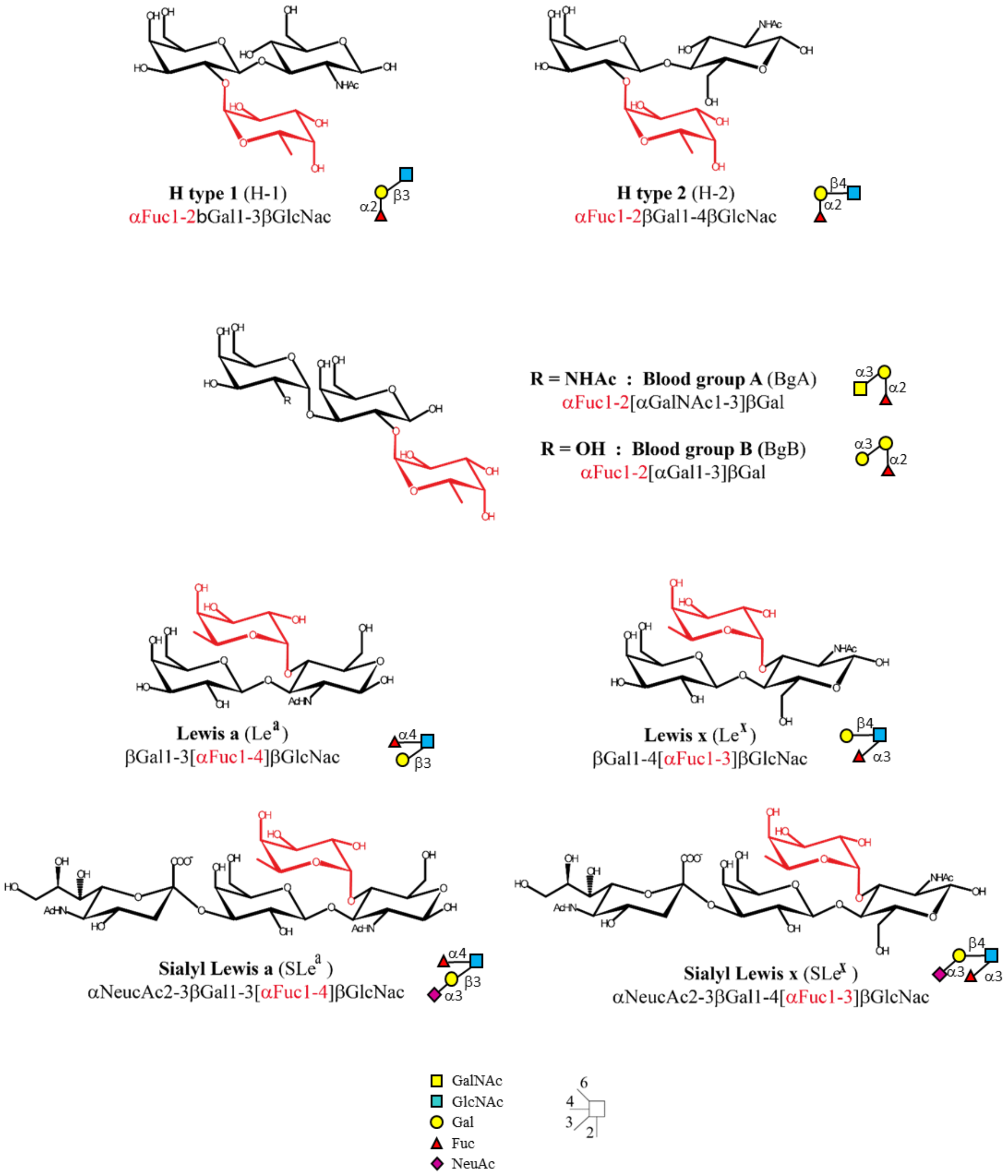

Supplementary Figure S5. Schematic representation of selected human fucosylated epitopes used for the specificity analysis of LecB. The fucose residue is colored in red in all representations.
