# Supplementary material for: Genomic Rearrangements and Functional Diversification of lecA and lecB Lectin-Coding Regions Impacting the Efficacy of Glycomimetics Directed against Pseudomonas aeruginosa
Source: Front Microbiol. 2016 May 31;7:811. doi: 10.3389/fmicb.2016.00811 (PMC4885879; doi:10.3389/fmicb.2016.00811)
Supplement: Supplementary file 14 [file Image6.PDF]

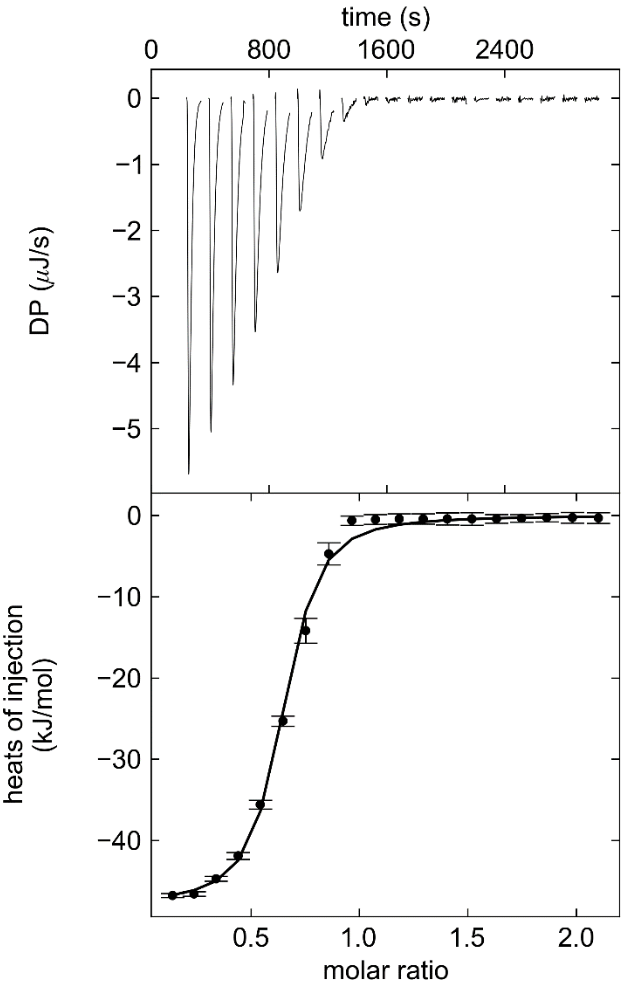

Supplementary Figure S6. Top. Raw ITC data obtained by injections of Lewis[a] tetrasaccharide (1.7 mM) in $LecB_{PA7}$ solutions (170μM). (Bottom) Corresponding integrated titration curve.
