# Supplementary material for: Genomic Rearrangements and Functional Diversification of lecA and lecB Lectin-Coding Regions Impacting the Efficacy of Glycomimetics Directed against Pseudomonas aeruginosa
Source: Front Microbiol. 2016 May 31;7:811. doi: 10.3389/fmicb.2016.00811 (PMC4885879; doi:10.3389/fmicb.2016.00811)
Supplement: Supplementary file 15 [file Image7.PDF]

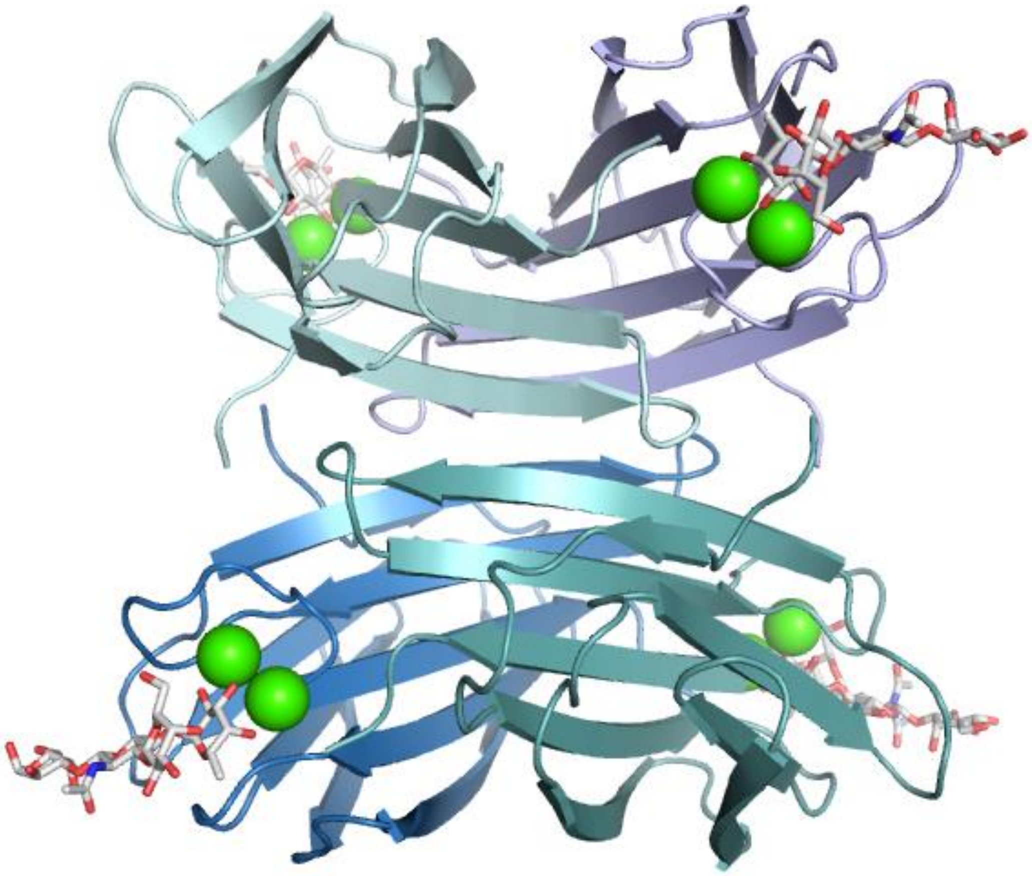

*Supplementary Figure S7.* Crystal structure of LecB$_{PA7}$ complexed with Lewis$^a$ tetrasaccharide. Each protein monomer is represented by a different color. Oligosaccharides are represented by sticks and calcium ions by spheres.
