# Supplementary material for: Genomic Rearrangements and Functional Diversification of lecA and lecB Lectin-Coding Regions Impacting the Efficacy of Glycomimetics Directed against Pseudomonas aeruginosa
Source: Front Microbiol. 2016 May 31;7:811. doi: 10.3389/fmicb.2016.00811 (PMC4885879; doi:10.3389/fmicb.2016.00811)
Supplement: Supplementary file 16 [file Image8.PDF]

(A)

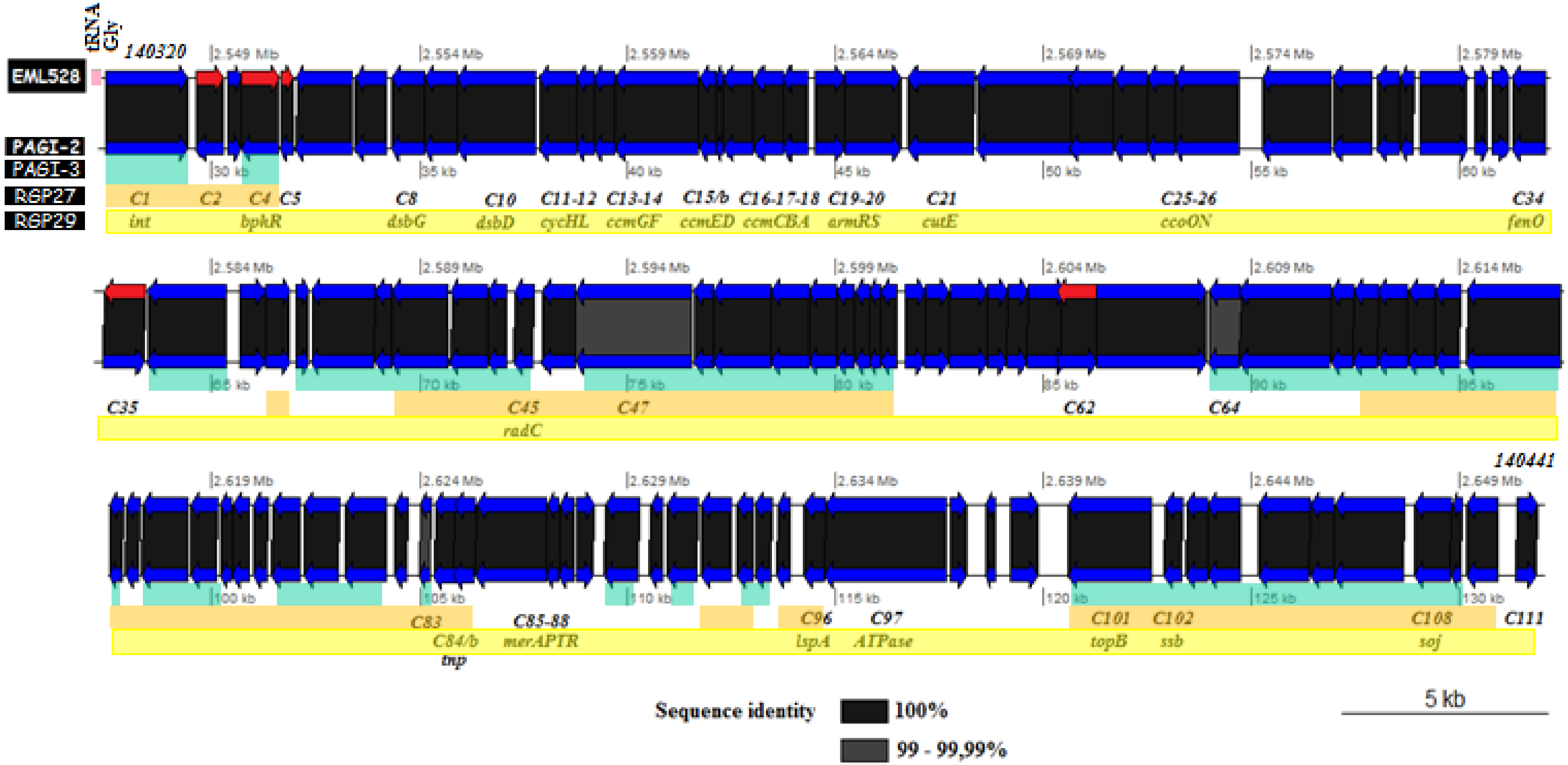

Supplementary Figure S8

(B)

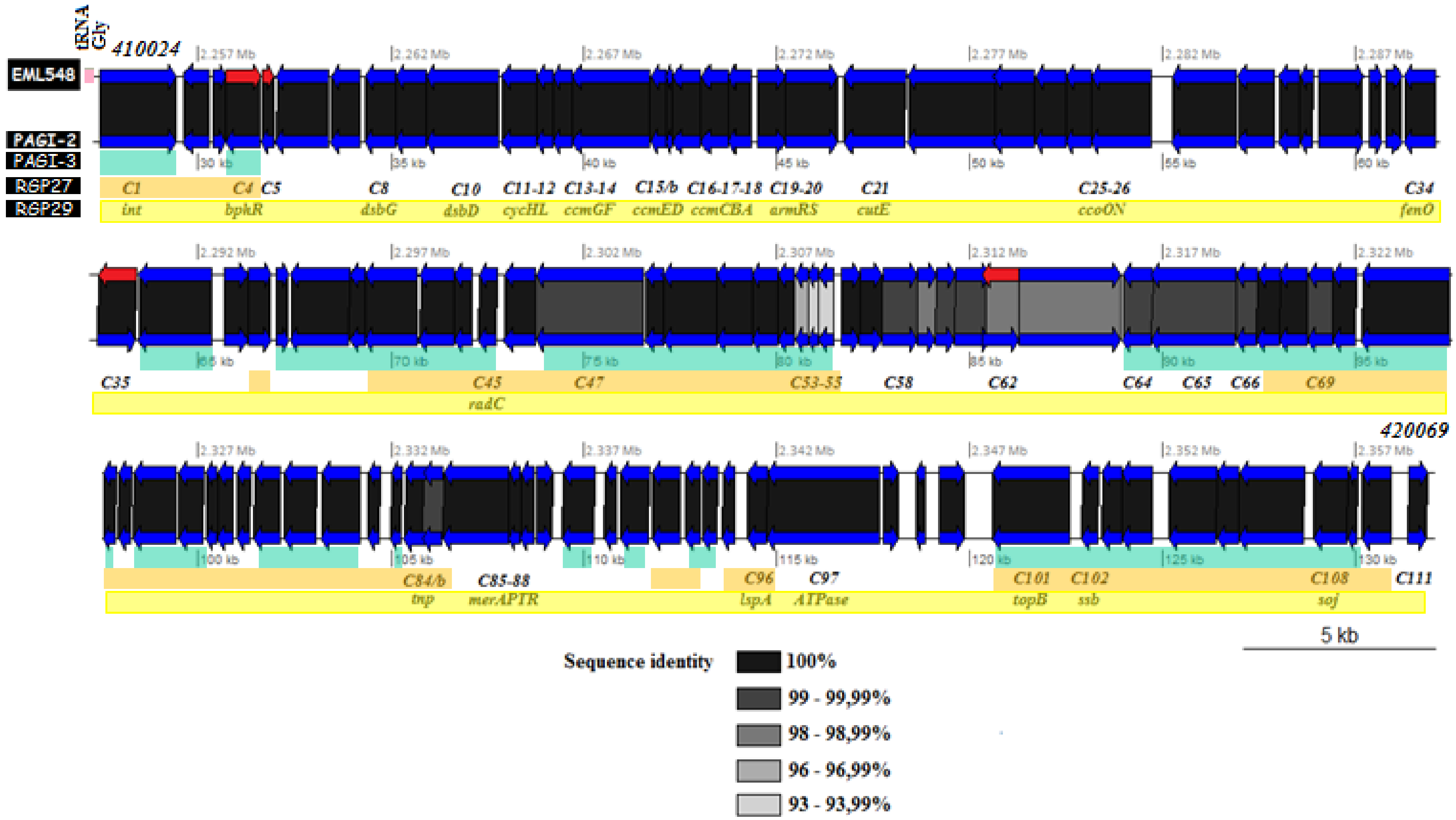

Supplementary Figure S8

Supplementary Figure S8. Blastn gene maps comparisons of the *P*. *aeruginosa* strains EML528 **(A)** and EML548 **(B)** PAGI-2 genomic islands. Predicted coding regions and their orientation are indicated by blue arrows (red if different from PAGI-2). $tRNA^{Gly}$ genes targeted for integration are indicated by a pink rectangle. CDSs detected in PAGI-2 (clone C, Larbig et al., 2002), PAGI-3 (strain SG17M, Larbig et al., 2002), RGP27 (strain PACS2, Mathee et al., 2008) and RGP29 (strain PA2192, Mathee et al., 2008) are represented by green, orange and yellow boxes, respectively. For full data on the CDSs functional categories see Larbig et al., (2002).
