# Supplementary material for: Genomic Rearrangements and Functional Diversification of lecA and lecB Lectin-Coding Regions Impacting the Efficacy of Glycomimetics Directed against Pseudomonas aeruginosa
Source: Front Microbiol. 2016 May 31;7:811. doi: 10.3389/fmicb.2016.00811 (PMC4885879; doi:10.3389/fmicb.2016.00811)
Supplement: Supplementary file 17 [file Image9.PDF]

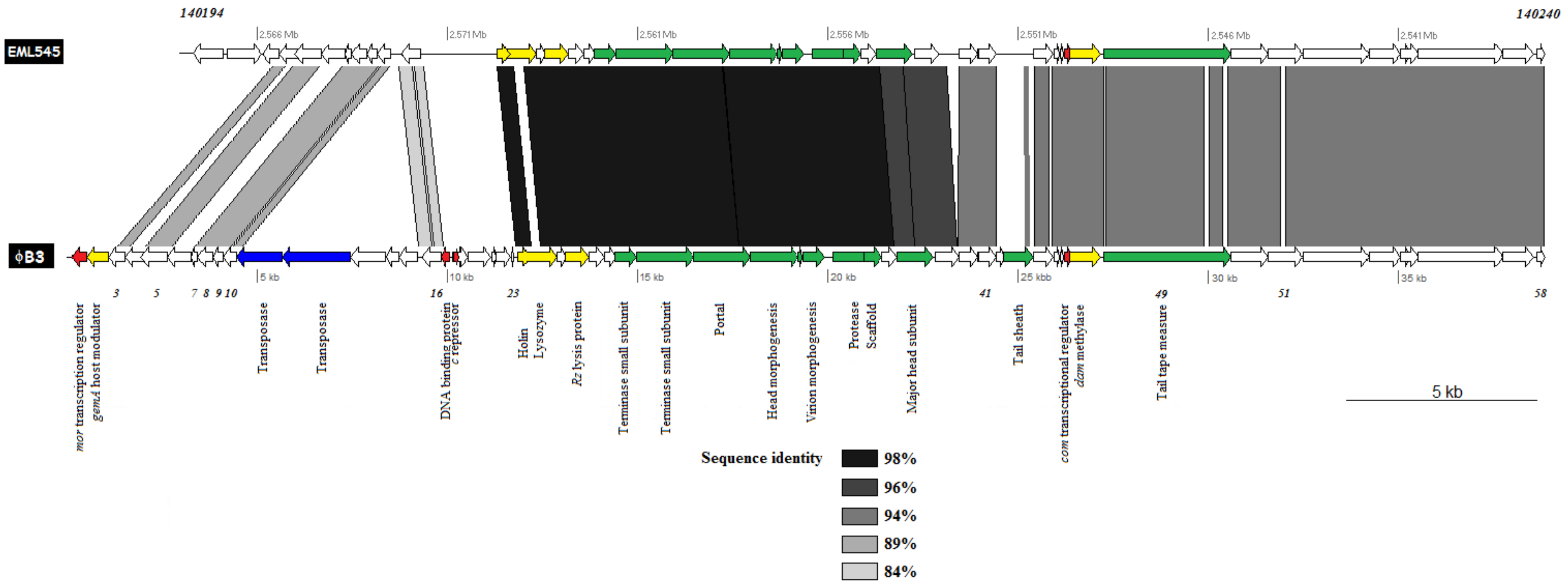

Supplementary Figure S9. Blastn gene maps comparisons of the *P*. *aeruginosa* strain EML545 variable *lecA* RGP90 harboring a bacteriophage B3 DNA sequence. Predicted coding regions are indicated by arrows showing their respective direction of transcription. Conserved CDSs involved in phage regulation are in red, those involved in integration and replication are in blue, those involved in host interaction in yellow, and the others in green or if hypothetical in white. CDSs encoding proteins with a predicted function are labeled. Sequence identities between DNA blocks are indicated. White regions indicate no identity between the analyzed sequences. Black lines in EML545 gene map indicate regions that were not annotated.
