# Supplementary material for: Genomic Rearrangements and Functional Diversification of lecA and lecB Lectin-Coding Regions Impacting the Efficacy of Glycomimetics Directed against Pseudomonas aeruginosa
Source: Front Microbiol. 2016 May 31;7:811. doi: 10.3389/fmicb.2016.00811 (PMC4885879; doi:10.3389/fmicb.2016.00811)
Supplement: Supplementary file 19 [file Image11.PDF]

(A)

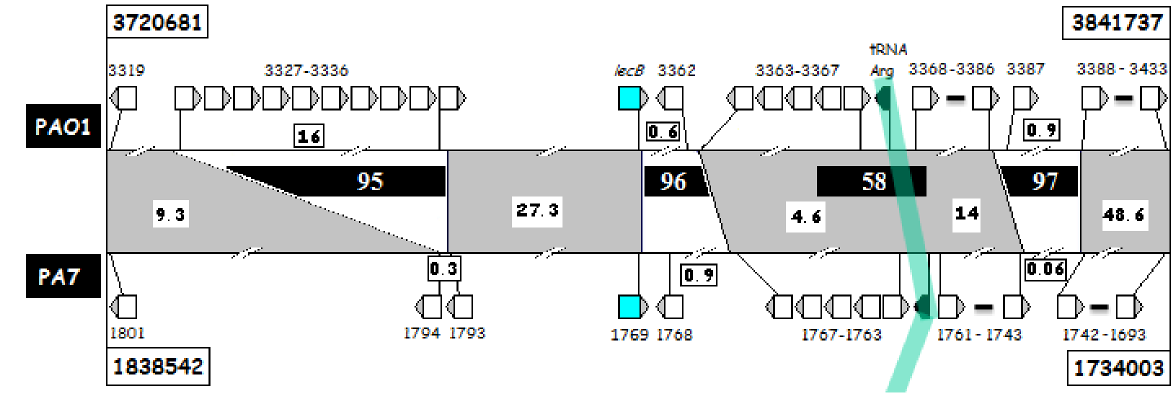

(B)

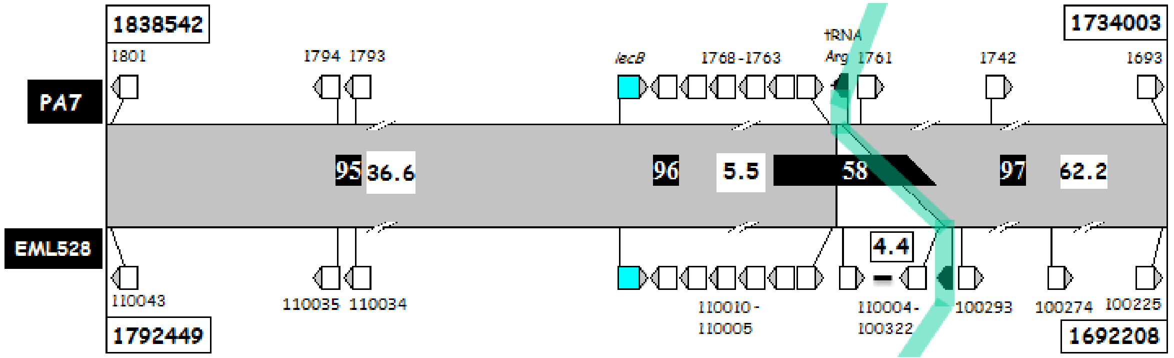

(C)

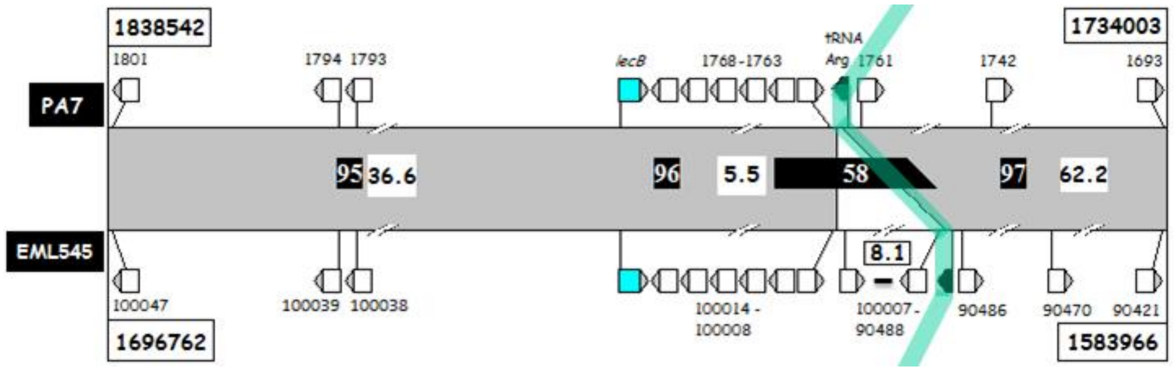

Supplementary Figure S11

(D)

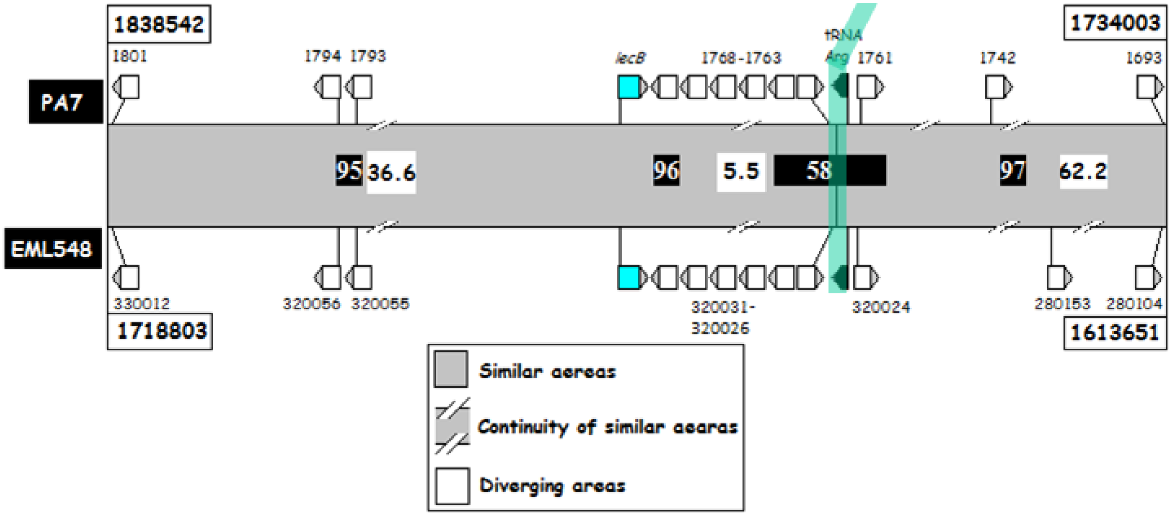

Supplementary Figure S11. ACT analyses of *lecB* genomic regions of *P. aeruginosa* strains PAO1, PA7, EML528, EML545 and EML548. **(A)** PAO1 (top) against PA7 (bottom); **(B)**, **(C)**, and **(D)**, PA7 (top) against, respectively, EML528 EML545 and EML548 regions (bottom). Horizontal black lines indicate the genome sequences compared over a length of about 120 kb. Grey indicates conserved regions, and white the variable ones. Distances between variable regions are given in kb. Regions of genomic plasticity (RGP) are indicated in black-boxes. Selected CDS (in white), and tRNA genes (in black) are shown. Orthologous tRNA genes between pairs of genomes are highlighted by a light-green background.
