# Supplementary material for: Genomic Rearrangements and Functional Diversification of lecA and lecB Lectin-Coding Regions Impacting the Efficacy of Glycomimetics Directed against Pseudomonas aeruginosa
Source: Front Microbiol. 2016 May 31;7:811. doi: 10.3389/fmicb.2016.00811 (PMC4885879; doi:10.3389/fmicb.2016.00811)
Supplement: Supplementary file 20 [file Image12.PDF]

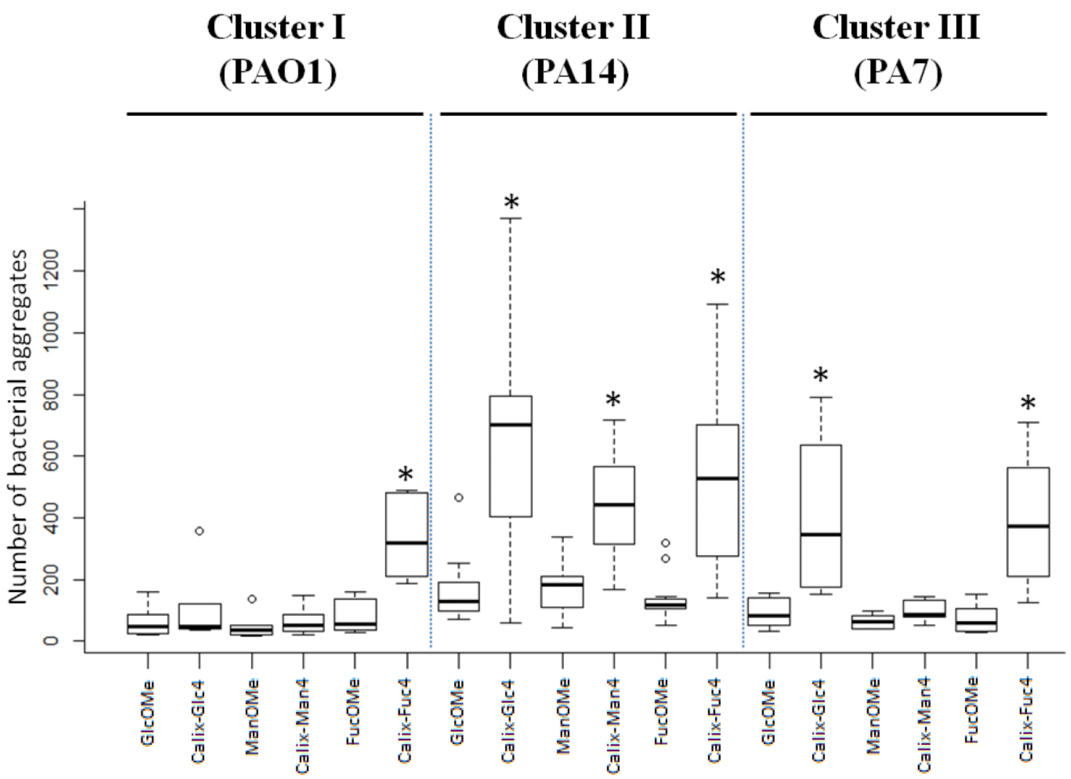

Supplementary Figure S12. Induction of *P. aeruginosa* cells aggregation by tetravalent calixarene glycoclusters (Calix-Glc_4, Calix-Man_4, Calix-Fuc_4 and Calix-Gal_4). Number of cell aggregates (>20 μm^2) were measured by FlowSight cytometry, and compared with those obtained after exposure to methyl glycosides (GlcOMe, ManOMe, FucOMe and GalOMe). These assays were performed on *P. aeruginosa* strains of LecB cluster I (PAO1, its isogenic mutants PAO1Δ*lecA* and PAO1Δ*lecB*, and bpoe1643), cluster II (PA14, bpoe1392, bpoe1737 and bpoe1748), and cluster III (PA7, EML528, EML545 and EML548) (according to **Figure 1**). Boxplots represent all values obtained for the tested strains according to these clusters. Aggregation levels were recorded from 20 000 cellular events. Data are expressed per mL. All tests were at least triplicated. Asterisks indicate significant differences between pairwise comparisons ($p < 0.05$).
